# Genome-wide association mapping and haplotype analysis reveal genetic architecture of seed fatty acid compositions in 1,550 diverse soybean accessions

**DOI:** 10.1186/s12870-025-07688-z

**Published:** 2025-11-25

**Authors:** Ahmed M. Abdelghany, Shengrui Zhang, Jing Li, Bin Li, Lijuan Qiu, Junming Sun

**Affiliations:** 1 The State Key Laboratory of Crop Gene Resources and Breeding, National Engineering Laboratory for Crop Molecular Breeding, MARA Key Laboratory of Soybean Biology (Beijing), Institute of Crop Sciences, Chinese Academy of Agricultural Sciences, 12 Zhongguancun South Street, Beijing, 100081 China; 2 Crop Science Department, Faculty of Agriculture, Damanhour University, Damanhour, Egypt

**Keywords:** Soybean (*Glycine max* [L.] merr.), GWAS, Fatty acid composition, Lipid biosynthesis, Haplotype analysis

## Abstract

Understanding the genetic architecture of soybean seed fatty acid (FA) compositions to enhance oil quality is crucial for nutritional value and industrial applications. This study elucidates the genomic determinants of seed FA composition in soybean (*Glycine max* [L.] Merr.) through comprehensive genome-wide association study (GWAS) analysis utilizing 1,550 diverse soybean accessions evaluated across five distinct environmental conditions. The phenotypic evaluation revealed significant genetic variability and environmental influences on the biosynthetic process of five essential FAs: palmitic (PA), stearic (SA), oleic (OA), linoleic (LA), and linolenic acid (LNA). High-throughput genomic association mapping identified 110,964 significant SNP-trait associations encompassing 18,841 putative genes. Notable genetic loci included chromosome 5 and 17 harboring *GmFATB1A* and *GmFATB1B* for PA biosynthesis; chromosome 2 and 8 containing *Glyma.02G161200* and *Glyma.08G279700* associated with SA regulation; chromosomes 10, 13, and 20 with *GmKCS21*, *GmKAS2*, and *GmFAD2* affecting OA concentration; chromosomes 10 and 13 with *GmKCS21* and *GmKAS2* influencing LA content; and chromosome 14 containing *GmFAD3* controlling LNA biosynthesis. Functional annotation through Gene Ontology (GO) and Kyoto Encyclopedia of Genes and Genomes (KEGG) pathway enrichment analyses revealed significant overrepresentation of lipid metabolic processes, particularly glycerolipid metabolic pathways. The haplotype characterization of three key regulatory genes *GmKCS21*, *GmFAD2*, and *GmFAD3* revealed distinct geographic distribution patterns across the northern region, Huang-Huai-Hai region, and southern ecoregions of China, with varying allelic frequencies between improved cultivars and landraces, reflecting adaptive evolution and selection pressure during domestication and enhancement. This study provides a comprehensive genetic resource of 110,964 SNP-trait associations and functionally characterized haplotypes of key regulatory genes (*GmKCS21*, *GmFAD2*, and *GmFAD3*) that demonstrate ecoregion-specific allele frequency patterns, enabling marker-assisted selection strategies tailored to those soybean production ecoregions.

**Supplementary Information:**

The online version contains supplementary material available at 10.1186/s12870-025-07688-z.

## Introduction

Soybean (*Glycine max* [L.] Merr.) is a globally cultivated crop due to its seeds’ high oil and protein content [1]. Soybean oil exhibits a specific fatty acid (FA) composition, comprising 16% saturated FAs, which includes 12% palmitic acid (PA) and 4% stearic acid (SA). Additionally, it contains 24% monounsaturated FAs, primarily oleic acid (OA), and 60% polyunsaturated FAs, with 53% linoleic acid (LA) and 7% linolenic acid (LNA) [2]. The functionality of soybean oil is dependent upon its FA composition.

Oils with high levels of saturated and monounsaturated FAs exhibit enhanced oxidative stability for culinary uses, while polyunsaturated FAs are prone to oxidation, resulting in the formation of undesirable flavor compounds [3]. From a nutritional perspective, the consumption of PA is associated with increased levels of low-density lipoprotein (LDL) cholesterol, whereas SA exhibits neutral effects on plasma LDL levels [4]. In contrast, unsaturated FAs are associated with lower concentrations of LDL cholesterol [5]. specifically, polyunsaturated FAs such as LA and LNA provide notable health advantages, potentially reducing the risk of cardiovascular disease and affecting cognitive function [6]. Furthermore, soybean oil, which is rich in these polyunsaturated FAs, plays a significant role in industrial applications, particularly as a drying oil component in paints and coatings [7, 8]. These FAs cannot be synthesized endogenously in humans and must be acquired through dietary sources.

FA composition in soybean demonstrates quantitative inheritance patterns influenced by a complex interaction of both major and minor genetic factors. Multiple studies have identified genetic loci that influence the five primary FAs using various methodologies [9–14]. Traditional quantitative trait loci (QTLs) mapping methods, utilizing bi-parental populations and linkage analysis, have primarily identified genomic regions significantly affecting FA profiles, especially where parental genotypes displayed considerable phenotypic divergence [15, 16]. These methodologies exhibit inherent limitations, including restricted allelic diversity limited to parental contributions and insufficient recombination events arising from constrained population sizes, which collectively lead to suboptimal mapping resolution.

An alternative approach to bi-parental linkage analysis is genome-wide association studies (GWAS), which utilize natural populations to exploit significantly enhanced recombination histories. The integration of genotyping by sequencing protocols, noted for their operational simplicity, methodological robustness, and economic efficiency, along with advancements in single nucleotide polymorphism (SNP) detection technologies, has significantly expanded the implementation of GWAS in soybean genomic research. Recent methodological advancements have facilitated the identification of genomic loci that governs various soybean seed quality composition including protein and oil contents [17–25].

Particularly, FA composition has also been examined through GWAS approach [9, 11, 13, 26–29], which provide an effective analytical framework for identifying genomic regions controlling FA traits by leveraging natural population structures and overcoming limitations inherent to those conventional bi-parental QTL mapping approaches. An investigation of 621 soybean accessions across five environments revealed 43 genomic regions that are significantly linked to FAs, corroborating previously identified loci, including *FATB1a*, *SACPD-C*, and *KAS3*, while also uncovering new regions associated with PA and SA [9]. Another comprehensive analysis of 290 diverse samples revealed 102 significant loci associated with FA components, with haplotype analysis identified key genes on chromosome 6 linked to SA content, where additional genes on chromosome 12 showed associated with OA, LA, and LNA [13]. Additional GWAS analysis involving 194 soybean accessions identified 149 SNPs distributed across 73 genomic regions and showed significant association with unsaturated FA contents, while expression analysis indicates that multiple genes may concurrently influence FA accumulation through various mechanisms [26]. However, the previous GWAS studies reveal several limitations: (1) The population sizes being examined are relatively small (ranging from 194 to 621 accessions), which may limit the statistical power needed to identify loci with minor effects and rare allelic variants; (2) The observed genetic diversity is constrained, potentially failing to capture the full spectrum of allelic variation present across different geographical origins and germplasm types; (3) There is a lack of integration of haplotype-based analyses with geographical distribution patterns, which impedes the understanding of evolutionary dynamics and breeding selection; and (4) There is insufficient evaluation across a range of environmental conditions, which is crucial for assessing genotype-by-environment interactions that affect FA composition.

To date, limited research has pinpointed candidate genes associated with seed FA composition in soybean by analyzing extensive germplasm collections. This study examined 1,550 diverse accessions, which included both cultivated varieties and landraces with varying domestication and breeding histories. Extensive genome-wide SNP markers were utilized across multiple environments, offering robust statistical power to identify genetic associations and evolutionary patterns influencing seed FA traits. Thus, this study aims to identify SNPs associated with five FA contents, explore potential genes in these regions and their roles in soybean FA metabolism, and analyze the distribution patterns of haplotypes of these candidate genes across various geographical origins, cultivars, and landraces to elucidate their evolutionary significance and differing effects on the five FAs.

## Materials and methods

### Plant material

A diverse panel of soybean germplasm, consisting of 1,550 accessions collected from 14 countries: China (1,285), United States (191), Russia (22), Japan (21), Brazil (8), Canada (6), Germany (4), South Korea (4), Eastern Europe (2), Italy (2), Thailand (2), Colombia (1), Nigeria (1), and North Korea (1). Detailed information for each accession can be found in Table S1. This panel of soybean germplasm was provided by the germplasm research group of the Institute of Crop Sciences, Chinese Academy of Agricultural Sciences (CAAS). The whole set of accessions in this study is conserved in the Chinese National Soybean Gene Bank (CNSGB).

### Field experiments and FA extraction and determination

Field trials were conducted in 2017 and 2018 at Changping (40°13′N, 116°12′E) in Beijing and Sanya (18°24′N, 109°5′E) in Hainan province, with additional trials conducted only in 2017 at Hefei (33°61′N, 117°0′E) in Anhui province. The experiments employed a randomized incomplete block design. The experimental design included single rows measuring 3 m in length, with an inter-row spacing of 50 cm and an intra-row spacing of 10 cm between plants. A randomized incomplete block design was utilized to plant the cultivars, with the different planting sites acting as replications. The cultivars were replicated across various locations owing to the extensive number of cultivars and the limited availability of land resources.

The five essential FAs—PA (C16:0), SA (C18:0), OA, (C18:1), LA (C18:2), and LNA (C18:3)—were derivatized to their methyl esters (FAMEs), following established methodologies [30] and their abundances determined by gas chromatography [31]. Prior to analysis, the GC system was calibrated using a five-point calibration curve constructed from FA standards (purity > 99%, Sigma-Aldrich) ranging from 0.1 to 5.0 mg/mL, with quality control standards analyzed every 15 samples to ensure measurement consistency and acceptable precision defined as coefficient of variation < 5%. Briefly, 20 g of seeds from each accession were finely ground with a Sample Preparation Mill (Retsch ZM100, Rheinische, Germany). Then, 300 mg of powder from each sample was weighed out using an analytic balance (Sartorius BS124S, Gottingen, Germany) and transferred to a 2-mL centrifuge tube preloaded with 1.0 mL n-hexane. This mixture was held for 20 min at 65 °C and shaken for 10 s every 5 min. Next, 1.0 mL sodium methoxide solution was added to the mixture, and it was shaken for 10 min on a twist mixer (TM-300, AS ONE, Osaka, Japan) at 65 °C to allow full methyl esterification of the FAs, followed by centrifugation at 12,000 ×g for 2 min. The supernatant was assayed to determine the concentrations of the methyl esters of the five FAs using a GC-2010 gas chromatograph (Shimadzu Inc., Kyoto, Japan) with flame ionization detector. The chromatographic separation was performed on an RTX-WAX column (Restek, Germany, 30 m length × 0.25 mm internal diameter × 0.25 mm thickness) and the temperature gradient was as follows: initially, the temperature was set at 180 °C for 1.5 min, then increased to 210 °C at a rate of 10 °C min^− 1^, held at 210 °C for 2 min, increased to 220 °C at a rate of 5 °C min^− 1^, and held at 220 °C for 5 min. The carrier gas was nitrogen, at a flow rate of 54 mL min^− 1^, and 1 µL of each sample was injected. Area was normalized to quantify the five FA concentrations using a GC2010 workstation [31].

### Association mapping and candidate gene prediction and annotation

Phenotypic and genotypic data from 1,550 diverse soybean accessions were utilized for GWAS to identify putative loci associated with FA contents across five individual environments (Hainan 2017, Hainan 2018, Beijing 2017, Beijing 2018, and Anhui 2017). Genotyping data for these accessions were obtained from a previously sequenced panel of 2,241 soybean lines [32], from which 1,550 accessions with complete phenotypic data were selected for this study. A total of 6,149,599 high-quality SNPs were utilized for GWAS after applying stringent quality control filters including: (i) minor allele frequency (MAF) ≥ 0.01, (ii) marker call rate ≥ 95%, and (iii) conformity to Hardy-Weinberg equilibrium expectations (*P* > 0.001). The filtered SNPs were uniformly distributed across the 20 soybean chromosomes with an average density of one SNP per 154.3 bp, providing comprehensive genome coverage for association mapping. Population structure was assessed using principal component analysis (PCA) to evaluate genetic stratification within the mapping population. The first two principal components collectively accounted for 40.47% of the total genetic variation, indicating moderate population structure among the accessions. To adequately control population stratification while maintaining statistical power, the first three PCs were incorporated as fixed effects in the association model.

Association mapping between genomic loci and the five FA traits was performed using mixed linear model (MLM) implemented in GAPIT v3.0 software [33]. The model incorporated a kinship matrix (K) calculated using the VanRaden method to account for genetic relatedness among accessions, while the first three PCs were included as fixed effects to correct for population stratification. The genome-wide significance threshold was determined using the Bonferroni correction method at *P* = 1 × 10⁻⁶ (equivalent to -log₁₀(*P*) ≥ 6), calculated as 1/6,149,599 to account for multiple testing across all SNPs. Model fitness was evaluated using quantile-quantile (Q-Q) plots, which compared expected versus observed P-values for each SNP to assess the extent to which significant associations exceeded random chance expectations. Manhattan plots were generated using GAPIT to visualize genomic associations for all five FA traits across the five environments as well as their overall average. Candidate gene prediction and functional annotation were conducted based on the soybean reference genome Wm82.a2.v1 using queries from the Phytozome (http://www.phytozome.net/soybean) and SoyBase (http://www.soybase.org/) databases.

Linkage disequilibrium (LD) between SNPs was assessed using pairwise correlation coefficients (r²) across all high-quality polymorphic sites. LD heatmaps were generated to visualize the correlation structure within and between candidate genomic regions. To characterize local LD patterns around the significant SNPs, LD decay was estimated as the physical distance over which *r²* decreased to half of its maximum value. Across the entire soybean panel, LD decayed to half-maximum at approximately 97 kb, consistent with previous reports [34, 35]. Accordingly, a ± 100 kb window around each lead SNP was used to define the candidate genomic region for gene identification, and genes located within these LD blocks (*r²* ≥ 0.8) were retrieved and annotated using the soybean reference genome (Wm82.a2.v1).

### GO and KEGG enrichment analysis

Gene ontology (GO) enrichment and Kyoto Encyclopedia of Genes and Genomes (KEGG) pathway analyses were performed on the 18,841 genes identified through GWAS. GO classifications were retrieved from the SoyBase database (http://soybase.org/) to determine over- and under-represented functional categories. Statistically significant GO terms across biological processes, cellular components, and molecular functions were further characterized using the PlantRegMap online platform (http://plantregmap.cbi.pku.edu.cn/go_result.php) [36]. Significance thresholds for GO enrichment were established at false discovery rate (FDR) < 0.05. Also, KEGG pathway enrichment analysis was conducted through the ShinyGO v0.60 online tool [37] to identify significantly overrepresented KEGG pathways. Visualization of both GO and KEGG analytical outputs was generated using RStudio (RStudio, Version 1.1.463, Inc., Boston, MA, USA).

### Haplotype analysis

The genetic sequences for the three candidate genes, *GmKCS21*, *GmFAD2*, and *GmFAD3*, were retrieved from the SoyFGB database (https://sfgb.rmbreeding.cn/index) for a total of 2214 soybean accessions in order to evaluate genetic diversity at these loci. SNPs were subjected to filtration criteria of MAF > 0.05. Due to substantial genotypic data absence, the examined populations were reduced to 999, 978, and 904 soybean accessions for the three respective genes. Analysis of FA composition was conducted under six different environmental conditions: Hainan 2017 (H17), Hainan 2018 (H18), Beijing 2017 (B17), Beijing 2018 (B18), Anhui 2017 (A17), in addition to the overall mean generated from these five unique environments.

## Results

### FA content analysis across different environments

A comprehensive analysis of FA composition was performed on the soybean germplasm collection overall and across the five environments studied. Overall, the findings of phenotypic analysis indicated that LA was the predominant FA, with a mean concentration of 54.23% and a low coefficient of variation (CV = 6.52%). For OA, it was the second most abundant FA, with a mean concentration of 21.63%, and exhibited significant phenotypic plasticity, as indicated by a CV of 19.52%. The average content of PA was 12.17%, exhibiting moderate variation (CV = 7.51%). In contrast, LNA and SA had mean values of 8.06% and 3.90%, while demonstrated significant variability, with CV of 18.40% and 15.31%, respectively. The descriptive statistics, heritability estimates (H²), and genotype × environment (GxE) interactions for these traits across the five environments are presented in Table S2.

Genetic analysis revealed high H² estimates across all five FAs, ranging from 0.68 to 0.84, indicating substantial genetic control of FA biosynthesis. Specifically, PA exhibited the highest heritability (H²=0.84), followed by LNA (H²=0.78), LA (H²=0.75), OA (H²=0.70), and SA (H²=0.68). Analysis of variance demonstrated statistically significant genotype-by-environment (G×E) interactions for all FA components, with mean square G×E values of 0.385 (*P* < 0.01) for PA, 0.259 (*P* < 0.05) for SA, 12.22 (*P* < 0.001) for OA, 7.618 (*P* < 0.001) for LA, and 1.284 (*P* < 0.01) for LNA. These significant G×E interactions indicate that genotype performance varied considerably across environments, suggesting that environmental factors modulate the genetic regulation of FA composition.

**Fig. 1:**
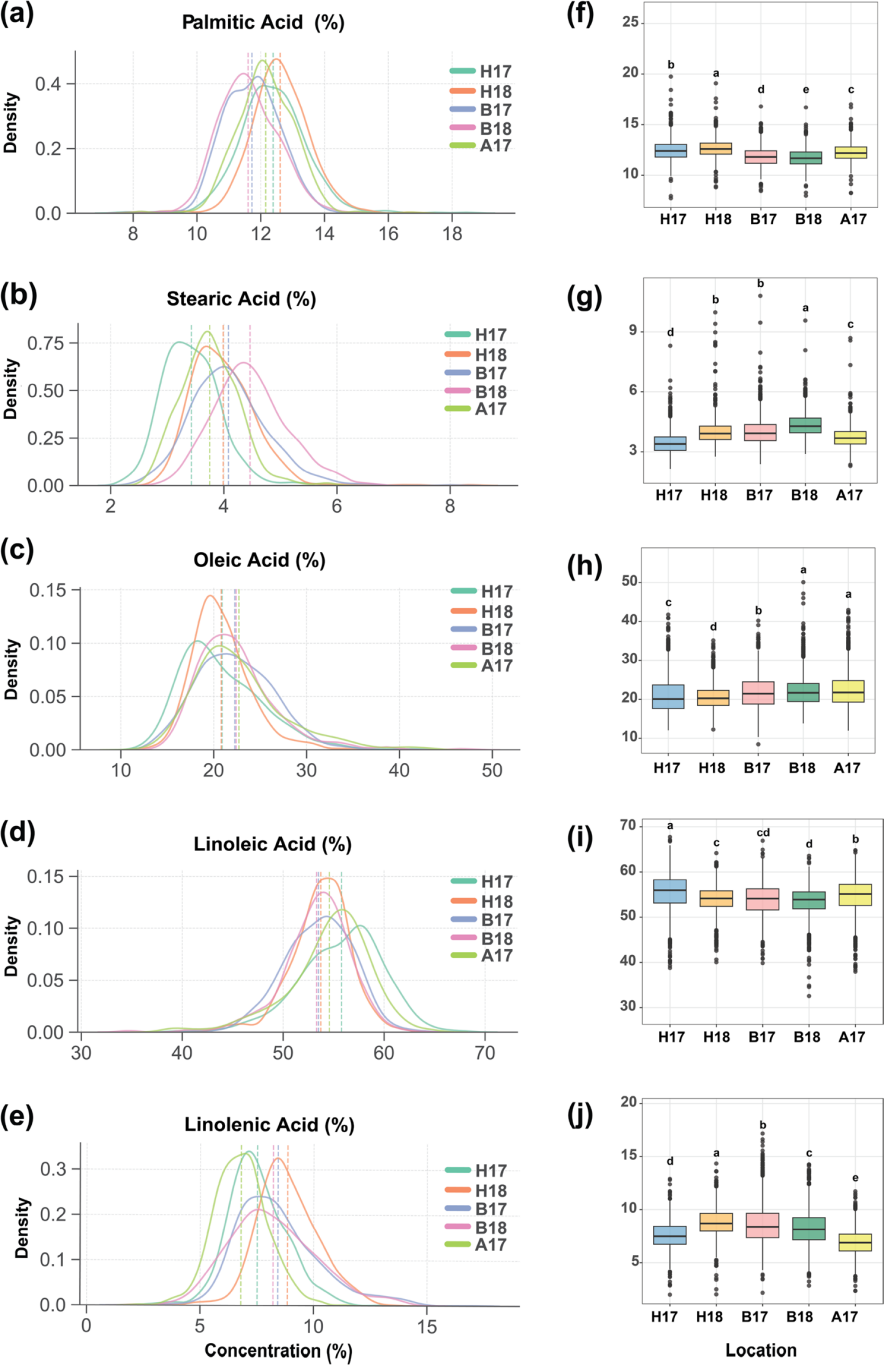
Distribution and variation of five FAs in soybean seeds across 1,550 accessions. **a-e** Normal distribution curves; (**f-j**) Boxplots of FA content across five environments: *H17* Hainan 2017, *H18* Hainan 2018, *B17* Beijing 2017, *B18* Beijing 2018, *A17* Anhui 2017. *PA* palmitic acid, *SA* stearic acid, *OA* Oleic acid, *LA* Linoleic acid, *LNA* Linolenic acid

The five FAs components exhibited approximately normal distribution patterns under all environmental conditions examined (Fig. 1a-e). The quantitative assessment of phenotypic variation parameters for individual FA constituents across distinct environments is also presented in Fig. 1f-j. Analysis of variance demonstrated statistically significant environmental effects on all FA components (*P* < 0.01), indicating pronounced genotype-by-environment interactions and substantial genetic diversity within the evaluated germplasm collection.

The concentrations of PA across five environments revealed statistically significant variations, with means ranging from 11.72% to 12.65% (Fig. 1f**)**. H18 exhibited the highest PA concentration (12.65%), which was significantly elevated compared to all other environments, while B18 displayed the lowest PA content (11.72%). All environments demonstrated statistically distinguishable PA levels as indicated by the distinct grouping designations (a-e). For SA, its concentrations across five environments revealed statistically significant variations, with means ranging from 3.44% to 4.36% (Fig. 1g**)**. Also, B18 exhibited the highest SA concentration (4.36%), which was significantly elevated compared to all other environments, while H17 displayed the lowest SA content (3.44%). B17 and H18 showed statistically similar SA levels (4.01% and 3.99%, respectively), while A17 exhibited an intermediate concentration (3.71%) that was significantly different from all other environments.

Analysis of OA concentrations demonstrated significant environmental influence, with means ranging from 20.63% to 22.50% (Fig. 1h**)**. A17 and B18 exhibited the highest OA concentrations (22.50% and 22.21%, respectively) with no significant difference between these environments. Conversely, H18 displayed the lowest OA content (20.63%). The concentration of LA exhibited significant environmental variation, with H17 showing the highest concentration (55.40%) and B18 the lowest (53.48%) (Fig. 1i**)**, whereas B17 and B18 demonstrated statistically similar LA levels (53.78% and 53.48%, respectively; groups cd and d). For LNA (Fig. 1j**)**, significant differences were observed across all environments, with concentrations ranging from 6.96 to 8.85%, while H18 exhibited the highest LNA accumulation (8.85%), while A17 displayed the lowest (6.96%). This pronounced environmental stratification for LNA suggests heightened sensitivity of ω−3 FA biosynthetic pathways to environmental factors compared to other unsaturated FAs.

### Genetic and environmental determinants of FA composition

**Fig. 2:**
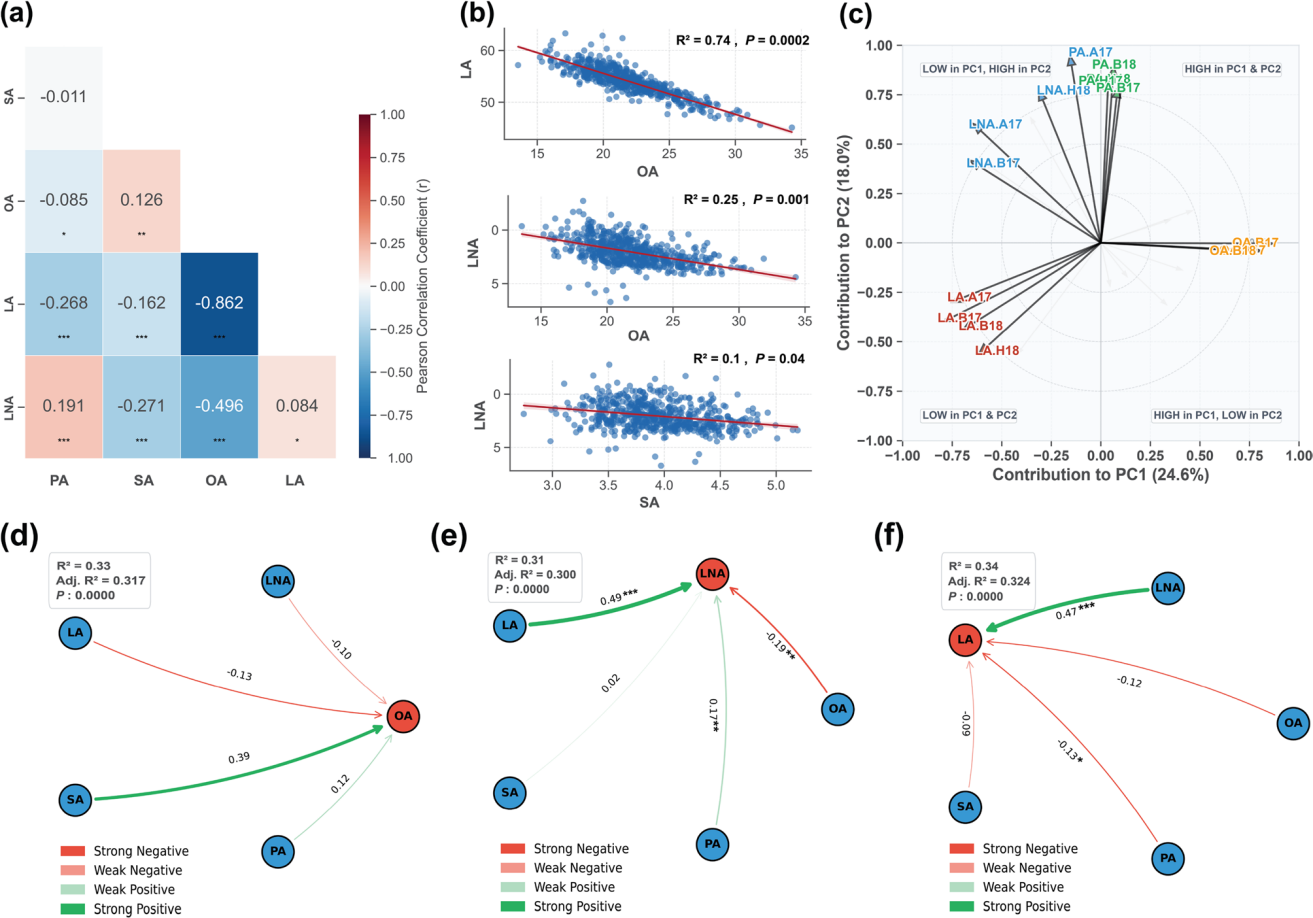
Comprehensive multivariate analysis of FA interactions in soybean seeds across multiple environments. **a** Correlation matrix revealing genetic correlations of FAs across five environments. **b** Scatter plots illustrate three key relationships of OA with LA, OA with LNA, and SA with LNA. (**c**) PCA plot demonstrates environmental and genetic variations in FA profiles. **d-f** Path coefficient analyses revealing direct effects of FAs on three key compositions of OA, LA, and LNA as response variables across different environments. *PA* Palmitic acid, *SA S*tearic acid, *OA O*leic acid, *LA *Linoleic acid, *LNA *Linolenic acid. Environments: H17, H18 (Hainan 2017 and 2018), B17, B18 (Beijing 2017 and 2018), A17 (Anhui 2017). Correlation significance: * *P* < 0.05, ** *P* < 0.01, *** *P* < 0.001

To further exploit the interrelationship among the five FAs, the complex interactions of FA composition in soybean seeds were revealed through comprehensive multivariate analyses (Fig. 2a-f). Correlation analysis showed intricate relationships among the five FAs, with particularly notable inverse correlations between LA and OA (Fig. 2a). These results were also confirmed by the pairwise scatter plot analyses, which demonstrated significant relationships between those key FAs, with the most pronounced correlation observed between OA and LA (*R²* = 0.74, *P* = 0.0002) (Fig. 2b). Such strong relationship between OA and LA suggests a fundamental metabolic trade-off in FA biosynthesis pathways. Additional correlations between OA-LNA and SA-LNA further illustrated the complex interdependencies in FA metabolism, even though with moderate correlation strengths (*R²* = 0.25, *P* = 0.001 and *R*² = 0.1, *P* = 0.04, respectively).

PCA plot provides a powerful visualization of FA composition variations across different soybean seed environments, explaining 42.6% of total metabolic variation, pinpointing the profound impact of environmental conditions on seed biochemistry (Fig. 2c). The biplot demonstrates significant metabolic plasticity, with distinct clustering of FAs like PA, LA and LNA, indicating how soybean genotypes dynamically adjust their FA synthesis in response to environmental conditions. It also captures the spatial and temporal diversity of FA profiles. Furthermore, the path coefficient analysis was also conducted, revealing intricate direct effects of some FAs on other key FAs in soybean seeds, which pinpoint the complex metabolic interactions across different environments (Fig. 2d-f). The findings of path analysis, with consistent model fits around *R²* = 0.31–0.34 and highly significant *P*-values, highlight the nuanced influential nature of saturated and unsaturated FAs on LNA, LA, and OA. The models reveal a sophisticated network of metabolic interactions, characterized by varying strengths of positive and negative pathways between different FAs.

### Population structure and linkage disequilibrium analysi

The phenotypic and genotypic data for 1,550 diverse soybean accessions were utilized for GWAS analysis to identify potential loci linked to FA contents across five distinct environments (Hainan 2017, Hainan 2018, Beijing 2017, Beijing 2018, and Anhui 2017). PCA was conducted to evaluate population stratification within the association panel. The analysis uncovered intricate genetic relationships among accessions, with landrace and improved cultivar groups exhibiting partial overlap, suggesting significant genetic variation within the collection (Fig. S1a). Distinct clustering patterns based on geographic origin were noted, with accessions from the Northern region (NR), Huang-Huai-Hai valley region (HR), Southern region (SR), and USA forming separate yet interconnected clusters. The initial two principal components (PCs) represented 40.47% of the overall genetic variation, indicating that population structure constituted a moderate share of the genetic diversity within the mapping population.

Analysis of genome-wide LD decay throughout the entire association panel showed that *r²* values decreased swiftly as physical distance increased (Fig. S1b). LD decreased to half of its maximum value at around 97 kb, reflecting a moderate level of LD that aligns with the varied genetic background of our soybean collection. Comparative LD analysis among subpopulations showed significant variations in LD decay rates. Enhanced cultivars demonstrated a slower rate of LD decay and longer haplotype blocks in comparison to landraces. Among geographic groups, USA accessions exhibited the most extensive LD patterns, whereas landraces from the Southern region (L_SR) demonstrated the most rapid LD decay, highlighting variations in selection intensity and genetic diversity across subpopulations. Considering these findings, we utilized a ± 100 kb window surrounding each lead SNP to pinpoint candidate genes located within high-LD regions (*r²* ≥ 0.8), thereby ensuring a robust linkage between associated markers and potential causal variants.

### GWAS reveal candidate loci underlying seed FA traits

We performed GWAS on FA composition utilizing a diverse set of 1,550 soybean accessions assessed across five distinct environments. The analysis of these traits was performed both within individual environments and through their averages to enhance the understanding of the genetic architecture associated with soybean seed FA composition. This comprehensive GWAS approach identified a total of 110,964 significant SNP–trait associations related to FA traits in soybean, spanning 18,841 genes across the 20 chromosomes (Table S3). Among the traits analyzed, SA exhibited the highest genetic complexity, with significant SNPs detected across multiple environments, ranging from 519 to 17,138 associations. Also, PA demonstrated substantial variability, with SNP counts ranging from 107 to 4,156 depending on the environmental condition. Other FA traits, including OA, LA, and LNA, exhibited trait-specific and environment-dependent patterns of genetic association. Notably, SA showed the most consistent genetic signal across environments, with thousands of SNPs frequently detected, yielding an average of 5,921 SNPs per environment. When averaged across all five environments, significant SNPs were detected for all FAs: PA (518 SNPs), SA (1,170 SNPs), OA (397 SNPs), LA (970 SNPs), and LNA (1,086 SNPs) (Table S3).

### Functional classification and metabolic pathway enrichment reveals lipid biosynthesis signatures in candidate genes

The genes identified via GWAS underwent gene ontology (GO) enrichment against the *G. max* genome using PlantRegMap. Of these 18,841 genes, 13,666 genes had GO annotations, yielding 223 significantly enriched terms (*P* < 0.05) (Table S4). These comprised 133 biological processes, 71 molecular functions, and 19 cellular components. Among biological processes, metabolic processes constituted the largest category (6,226 genes), followed by cellular processes (4,483 genes). Within the cellular component category, cell and cell part represented the largest subcategory (3,630 genes). For molecular function, biological activities constituted the predominant subcategory (14,497 genes), followed by binding (2,037 genes). Five lipid-related processes were among the significantly enriched GO terms: glycerolipid biosynthetic process, phosphatidylglycerol metabolic process, phosphatidylglycerol biosynthetic process, lipid metabolic process, and triglyceride biosynthetic process.

**Fig. 3:**
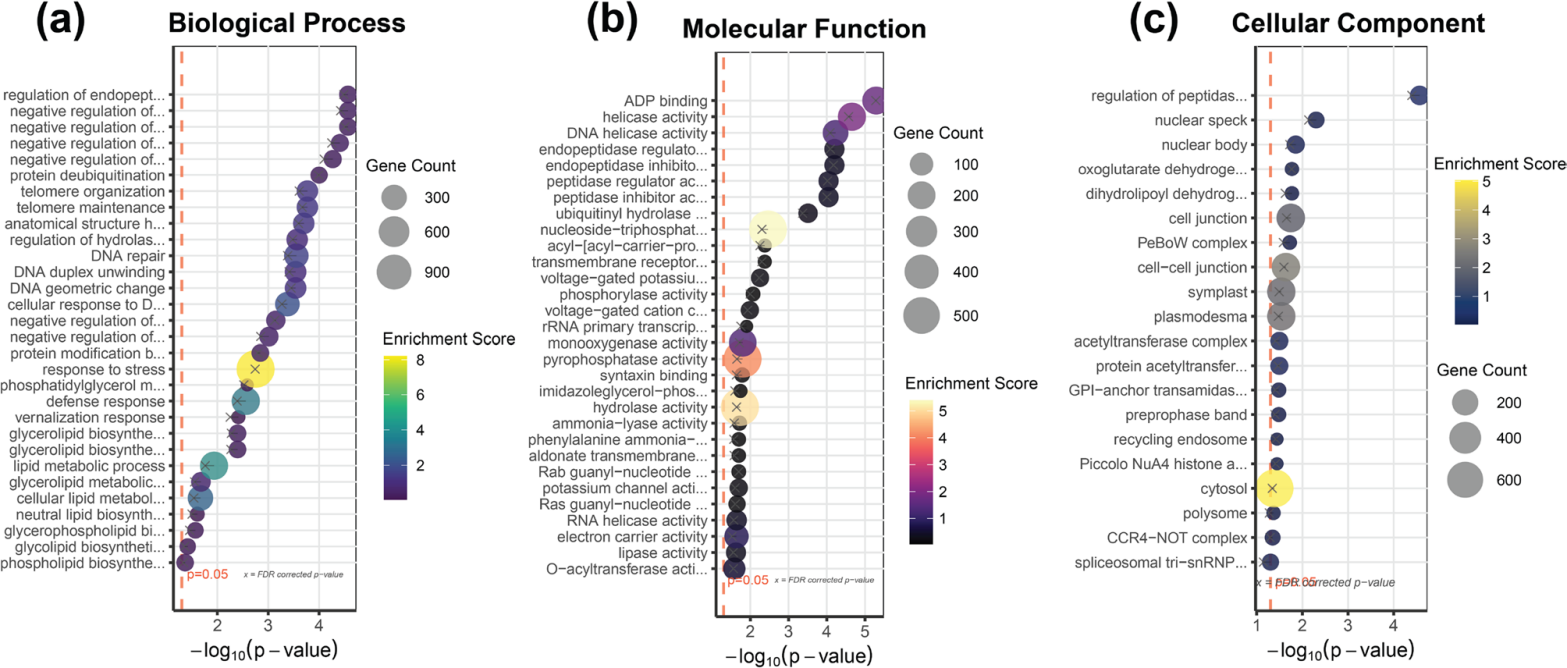
Top significantly enriched Gene Ontology (GO) terms among 18,841 GWAS-identified genes. The bubble plots display significantly enriched GO terms in three categories: (**a**) Biological process terms, (**b**) Molecular function terms, (**c**) Cellular component terms. The x-axis represents statistical significance as -log10(*P*-value), with the red dashed line indicating the significance threshold (*p* < 0.05). Bubble size corresponds to gene count (number of genes associated with each term), while color intensity reflects enrichment score. Yellow “x” markers identify notable terms with distinctive functional importance.

To further indicate the functional annotation specificity, top significant GO terms indicating the highest significantly enriched GO terms in each of the three go categories comprising 30 biological processes, 30 molecular function, and the whole 20 cellular component GO terms were visualized (Fig. 3). The figure highlighted glycerolipid biosynthetic process as a key biological process, while acyl-[acyl-carrier-protein] desaturase activity emerged as the most significant molecular function. Acyl carrier protein desaturase (ACP) genes, particularly stearoyl-acyl carrier protein desaturase (SAD), catalyze the initial desaturation step producing OA, which serves as a precursor for LA and LNA, thus critically influencing unsaturated FA content.

**Fig. 4:**
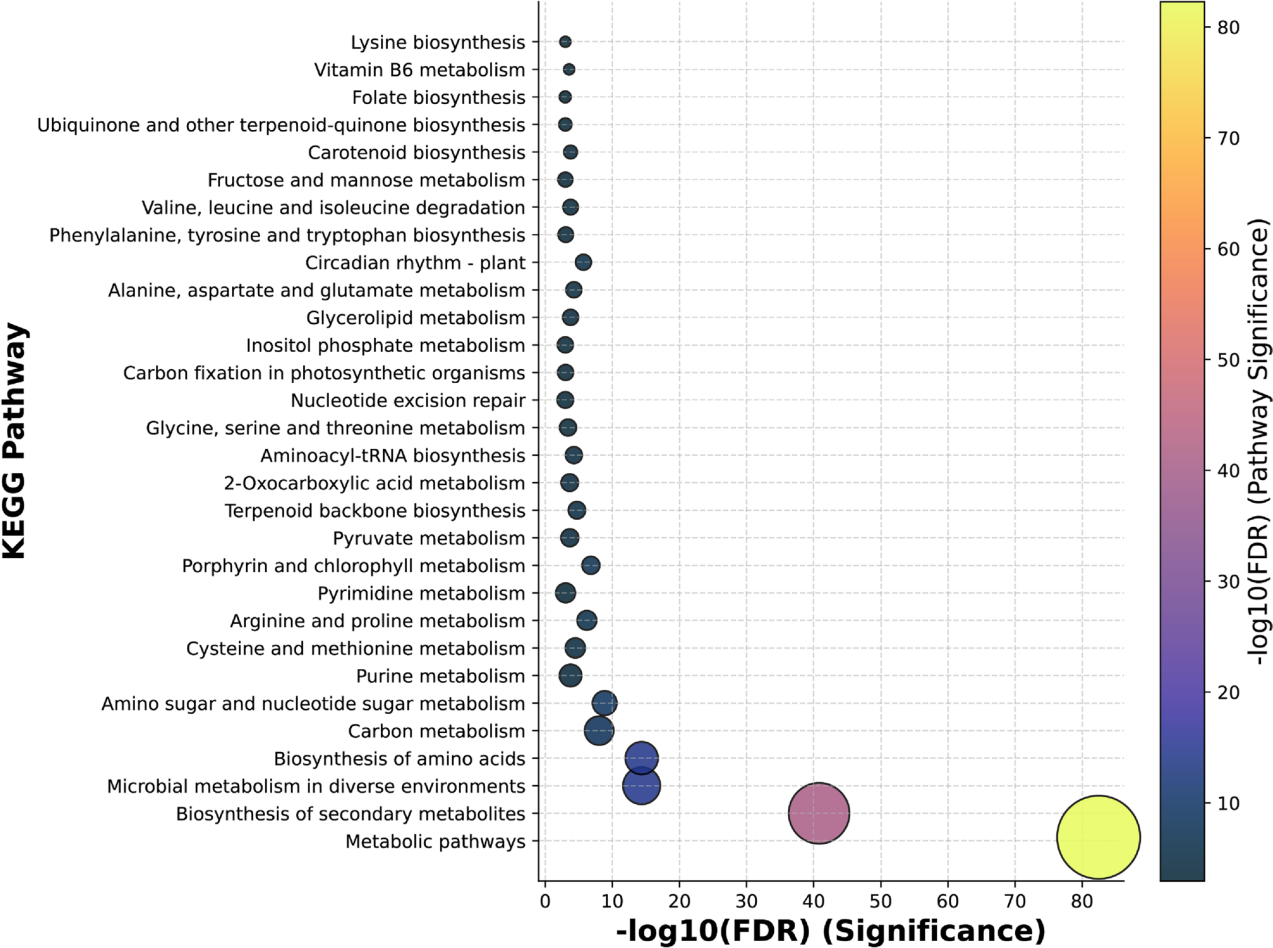
KEGG pathway enrichment analysis of 18,841 GWAS-identified genes. The bubble plot displays significantly enriched pathways, with the x-axis representing statistical significance as -log10(FDR) values and bubble size corresponding to the number of genes associated with each pathway. Color intensity indicates pathway significance, with yellower colors representing higher significance levels

Kyoto Encyclopedia of Genes and Genomes (KEGG) pathway analysis was performed to characterize the functional distribution of the 18,841 GWAS-derived genes. KEGG pathway annotations, widely utilized as a reference for candidate gene screening, revealed metabolism as the most enriched pathway category. The predominant pathways with direct relevance to oilseed traits included glycerolipid metabolism, biosynthesis of secondary metabolites, biosynthesis of amino acids, carbon metabolism, and amino sugar and nucleotide sugar metabolism (Fig. 4, Table S5). Notably, glycerolipid metabolism, the primary focus of this study, ranked among the most significantly enriched KEGG pathways, validating the relevance of our GWAS findings.

### GWAS reveals key genetic factors controlling FA compositions in soybean

**Fig. 5:**
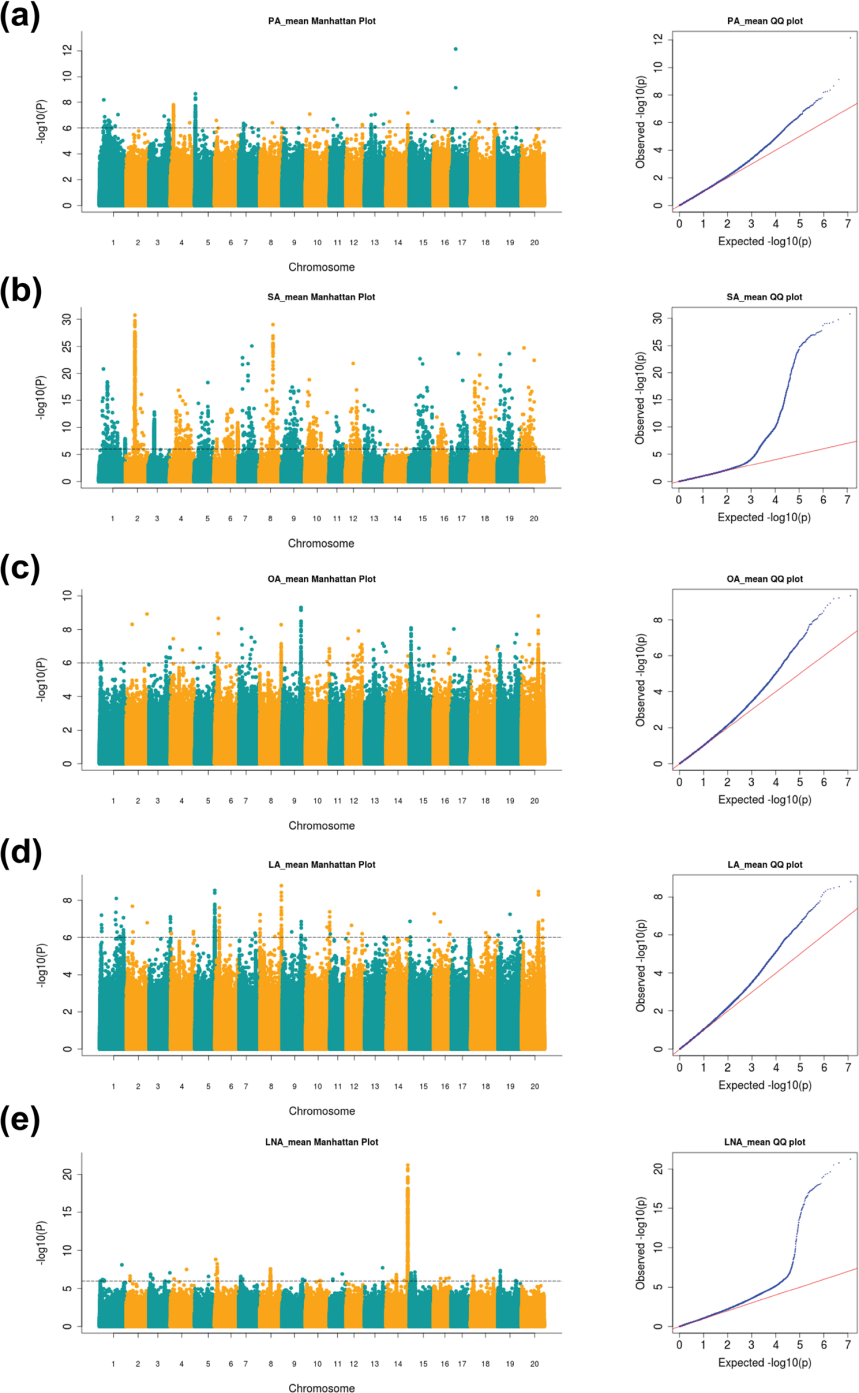
GWAS Manhattan and Q-Q plots for seed FA traits in soybean, derived from phenotypic data averaged over five environments. The left panels exhibit Manhattan plots that depict -log10(*P*) of peak values against chromosomal locations, whereas the right panels present equivalent quantile-quantile (Q-Q) plots illustrating observed vs. expected -log10(P) distributions. **a** *PA* Palmitic acid, (**b**) *SA *Stearic acid, (**c**) *OA* Oleic acid, (**d**) *LA* Linoleic acid, and (**e**) *LNA* Linolenic acid. Adjacent chromosomes are distinguished by alternating blue and orange coloring. The horizontal grey dashed line denotes the genome-wide significance criterion (*P* < 1 × 10^−6)

The findings of GWAS revealed multiple genomic regions controlling FA composition in soybean (Table 1**)** for the overall mean across the five environments (Fig. 5a-e**)** and for each of the five environments (Fig. S2a-e). The statistical validity of our genetic analysis was confirmed by Q-Q plots, demonstrating a strong harmony between expected and observed genetic associations.

**Table 1:** Candidate genomic regions and associated genes for soybean seed FA traits revealed by GWAS approach in this study

Trait	Chr.	Candidate Region			Length (bp)	No. SNP	Peak value	Matched_QTL	No. Identified Genes	Candidate Genes
		Start (bp)		End (bp)						
PA	5	950,673		1,291,694	341,022	1663	8.6663895	-	29	*Glyma*.05G012300
	17	9,520,135		9,720,772	200,638	32	12.137931	Seed palmitic plus stearic 1–1, Seed palmitic 4 − 3, Seed linoleic 6–6	16	*Glyma*.17G120400
SA	2	19,670,066	20,097,733		427,668	1092	30.78129	Seed linoleic 7 − 1, Seed linolenic 6 − 3, Seed linolenic 7 − 3	4	*Glyma*.02G161200
										*Glyma*.02G161300
										*Glyma*.02G161400
	8	37,833,470	38,105,696		272,227	27	14.74195	Seed linoleic 6 − 2	9	*Glyma*.08G279700
OA	10	50,840,669	51,052,625		211,957	104	6.848809	Seed linoleic 6–7	26	*Glyma.10G291700*
	13	22,511,266	22,739,284		228,019	368	9.349129	Seed linoleic 6 − 3	23	*Glyma*.13G112700
	20	544,187	1,026,236		482,050	1311	8.015971	Seed oil 42 − 36	58	*Glyma*.20G007900
		18,433,866	18,633,866		200,001	3	9.162897	Seed palmitic 10 − 2	3	*Glyma*.20G060100
										*Glyma*.20G060300
		35,118,060	35,591,062		473,003	1188	8.8155041	Seed linoleic 6–8, Seed palmitic 10 − 2, Seed oil 27 − 4	54	*Glyma.20G111000*
LA	10	50,840,669	51,052,625		211,957	182	7.377091	Seed linoleic 6–7	26	*Glyma.10G291700*
	13	22,511,266	22,738,545		227,280	322	10.00705	Seed linoleic 6 − 3	23	*Glyma*.13G112700
LNA	14	45,804,356	46,085,528		281,173	161	21.23388	Seed oil 43 − 5, Seed linolenic 3–3, Seed linolenic 10 − 2, Seed linoleic 3–3	3381	*Glyma.14G194300*

For PA, two significant regions were identified on Chromosome 5 and 17 (Fig. 5a**)**. The region (950673–1291694 bp) in Chromosome 5 contained 1,663 SNPs with a peak association signal of -log₁₀(*P*) = 8.67, encompassing 29 candidate genes. Among these, *Glyma.05G012300*, which encodes a palmitoyl-acyl carrier protein thioesterase (*FATB1A*) in chloroplast involved in the FA biosynthesis pathway, was identified. This gene spans an interval of 1,127,438–1,131,632 bp and contains 57 SNPs and was consistently detected across multiple environments, specifically in H18 and H17, as well as in average of the five environments. The Chromosome 17 region (9520135–9720772 bp) harbored 32 SNPs with a peak association signal of -log₁₀(*P*) = 12.14 and co-localized with QTLs for Seed palmitic plus stearic 1–1, seed palmitic 4 − 3, and Seed linoleic 6–6. This region contained 16 candidate genes, including *Glyma.17G120400* (9570425–9574806 bp), which also encodes palmitoyl-acyl carrier protein thioesterase (*GmFATB1B*). This gene functions in FA biosynthesis and contains 2 SNPs associated with the trait. The encoded enzyme performs analogous catalytic activity to the gene product identified on Chromosome 5, potentially indicating functional redundancy within the PA biosynthetic pathway.

Regarding SA content, two regions were also detected showing significant association, including one region on Chromosome 2 and another candidate one on Chromosome 8 (Fig. 5b). The candidate region on Chromosome 2 (19,670,066–20,097,733 bp) spanned 427,668 bp with 1,092 SNPs and displayed the highest association signal (30.78) among all detected loci. This region overlapped with previously reported QTLs including Seed linoleic 7 − 1, Seed linolenic 6 − 3, and Seed linolenic 7 − 3, and contained three candidate genes: *Glyma.02G161200*, *Glyma.02G161300*, and *Glyma.02G161400*.

Notably, *Glyma.02G161200*, which encodes a thioredoxin-like 3 − 2, chloroplastic protein, emerged as a promising candidate. This gene spans from 19,687,330 to 19,695,623 bp and harbored 273 SNPs, a remarkably high density relative to gene length. Importantly, these SNPs were consistently detected across all five studied environments, and the gene showed a strong average association signal across environments, reinforcing the stability and robustness of its potential effect on SA content. Functionally, *Glyma.02G161200* is homologous to *Arabidopsis thaliana WCRKC2* (UniProt ID: Q8VZT6), a thioredoxin-like protein localized in the chloroplast. While thioredoxins are not directly involved in FA synthesis, they play essential roles in redox regulation of plastidic enzymes. Enzymes such as acetyl-CoA carboxylase (ACCase) and ketoacyl-ACP synthases (*KAS*) are known to be redox-sensitive and subject to post-translational regulation mediated by thioredoxins. Collectively, the strong genetic association, chloroplastic localization, and known regulatory function suggest that *Glyma.02G161200* may influence SA content by modulating the redox state of key biosynthetic enzymes in the chloroplast. Its consistent detection across environments and co-localization with established QTLs may support its candidacy as a regulatory gene involved in chloroplastic FA biosynthesis.

The second region was located on chromosome 8, covering a segment of 272,227 bp from position 37,833,470 to 38,105,696, containing 27 significant SNPs with a peak association signal of -log₁₀(*P*) = 14.74. This region overlapped with a previously reported QTL, Seed linoleic 6 − 2, and contained nine candidate genes. Notably, *Glyma*.*08G279700*, which encodes a very-long-chain (3R)−3-hydroxyacyl-CoA dehydratase involved in the FA elongation pathway, emerged as a promising candidate. This gene harbored 3 SNPs that were consistently detected across three different environments (B18, H17, and H18) as well as in the overall average across all five environments.

**Fig. 6:**
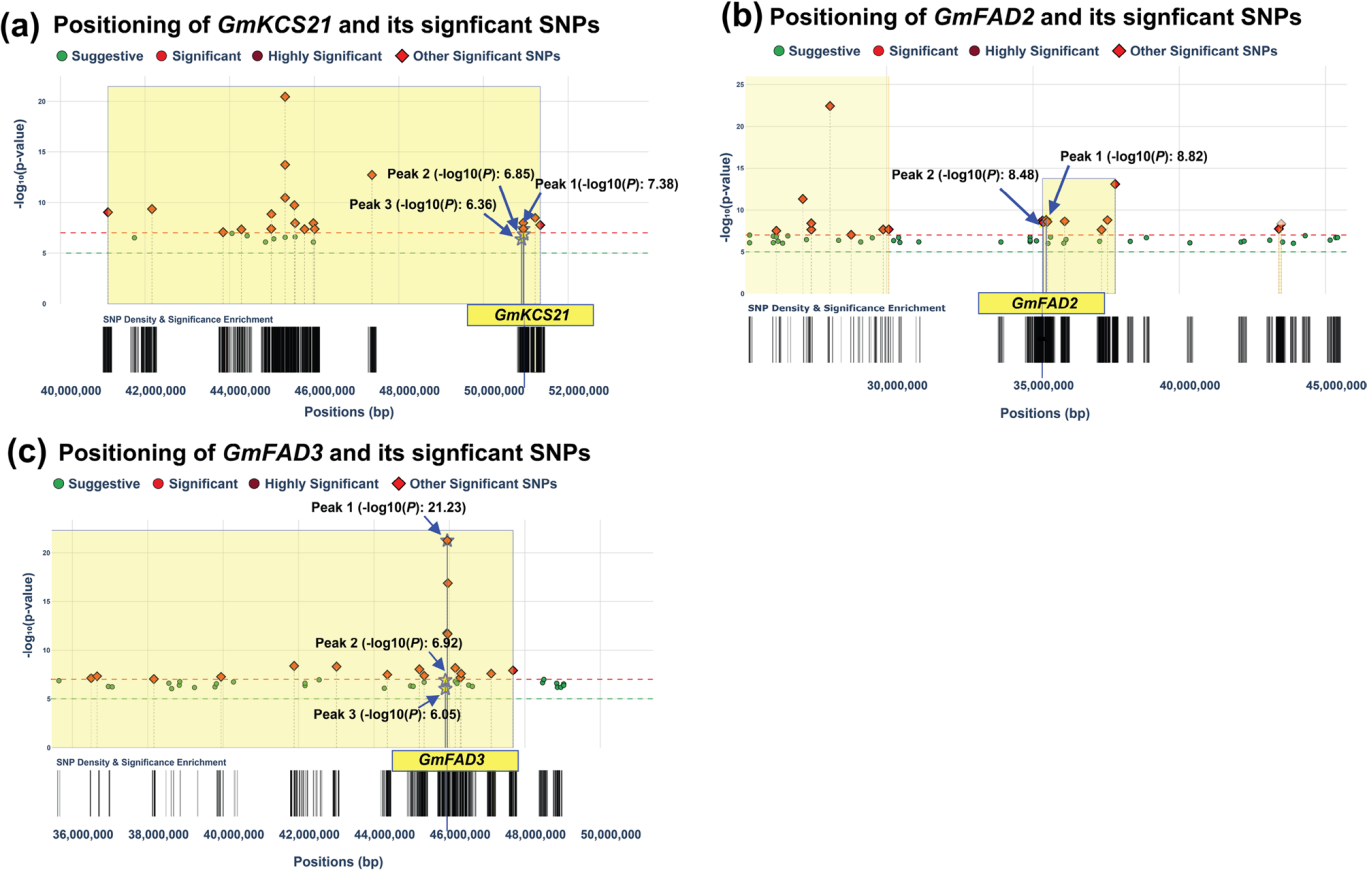
Visualization of genomic data highlighting significant SNPs linked to FA traits within three candidate genes. **a** *GmKCS21* (*Glyma.10G291700)* region exhibits three notable peaks with -log_10_(*P*) values of 7.38, 6.85, and 6.36. **b** *GmFAD2* (*Glyma.20G111000*) region displays a highly significant peak with a -log_10_(*P*) value of 8.48, accompanied by another notable peak value of with a -log_10_(*P*) = 8.82. **c** *GmFAD3* (*Glyma.14G194300*) region shows an exceptionally significant peak with a -log_10_(*P*) value of 21.23, accompanied by two additional peaks with -log_10_(*P*) values of 6.92 and 6.05

Five genomic loci, distributed across three chromosomes, demonstrated statistically significant association with OA content (Fig. 5c**)**. The region spanning chr10:50840669–51,052,625 exhibited particularly strong statistical evidence, harboring 104 significant SNPs with peak association value of -log₁₀(*P*) = 6.85. This genomic interval, co-localizing with the previously identified Seed linoleic 6–7 QTL, encompasses 26 annotated genes, with *Glyma.10G291700* (chr10:50964178–50966466) identified as promising candidate due to its role in encoding 3-ketoacyl-CoA synthase 21 (*KCS21*), also referred to as *GmKCS21*, an enzyme essential to the FA elongation pathway. Notably, *GmKCS21*, in this study, exhibited significant associations with multiple FA traits, as evidenced by the genomic analysis results. Specifically, this locus harbored seven SNPs significantly associated with LA, demonstrating a peak association value of -log₁₀(*P*) = 7.38. Additionally, the same genetic locus contained two SNPs strongly associated with SA with a peak value of -log₁₀(*P*) = 6.36 and four SNPs associated with OA with a peak value of -log₁₀(*P*) = 6.848 (Fig. 6a).

The second significant region was detected on Chr13 (chr13:22511266–22739284) containing 368 SNPs with a peak association value of -log₁₀(*P*) = 9.35, which co-localizes with the previously characterized Seed linoleic 6 − 3 QTL. Within this genomic interval, we identified 23 genes, with *Glyma*.*13G112700* (chr13:22511266–22739284) emerging as the primary functional candidate based on its direct involvement in FA biosynthesis. This gene encodes ketoacyl-ACP synthases 2 (*KAS2*), a chloroplast-localized enzyme that catalyzes a crucial condensation reaction in the type 2 FA biosynthesis pathway. The association signal for this locus contained 16 significant SNPs and demonstrated environmental specificity, with significant associations detected exclusively in environments B17 and B18.

The remaining three significant regions were identified on Chr20 (chr20:544187–1026236, chr20:18433866–18633866, and chr20:35118060–35591062), spanning 482,050 bp, 200,001 bp, and 473,003 bp, and containing 1,311, 3, and 1,188 significantly associated SNPs, respectively. These regions co-localize with several previously identified QTLs, including Seed oil 42 − 36, Seed palmitic 10 − 2, Seed linoleic 6–8, and Seed oil 27 − 4. Among these three regions, the interval at chr20:35118060–35,591,062, which co-localizes with Seed linoleic 6–8, Seed palmitic 10 − 2, and Seed oil 27 − 4 QTLs, is particularly noteworthy as it contains 54 candidate genes, including *Glyma.20G111000*, which harbors 22 SNPs with a peak association value of -log_10_(*P*) = 8.82, being staple across three environments, including B17, B18, and H17, in addition to the overall average of the five environments. This gene encodes the Omega-6 FA desaturase (*GmFAD2*), an endoplasmic reticulum-localized enzyme that catalyzes the desaturation of OA (C18:1Δ9) to LA (C18:2Δ9,12), representing a critical control point in the FA biosynthesis pathway that directly influences the balance between monounsaturated and polyunsaturated FAs in seed oil. Noteworthy, *GmFAD2*, in this study, exhibited significant associations with two FA traits (OA and LA) as shown in Fig. 6b. This locus contained 15 SNPs significantly associated with LA with a peak value of -log_10_(*P*) = 8.48, and 22 SNPs associated with OA with a higher peak value of -log_10_(*P*) = 8.82. highlighting its crucial role in regulating the balance between mono- and polyunsaturated FAs in soybean.

For LA-significantly associated regions, two significant genomic regions were identified (Fig. 5d). The first region, located on chromosome 10 (chr10:50840669–51052625), encompassed 182 SNPs with a maximum association significance value of -log_10_(*P*) = 7.38. This region demonstrates spatial coincidence with the previously characterized seed linoleic 6–7 QTL. This region encompasses 26 annotated genes, including *Glyma.10G291700 (GmKCS21)*. The association signal at this locus demonstrated environmental stability, with significant associations detected across both environments B17 and H17, as well as in the multi-environment average analysis. The second region on chromosome 13 (chr13:22511266–22738545) harbored 322 significantly associated SNPs with a peak value -log_10_(*P*) = 10.01 and overlapped with the seed linoleic 6 − 3 QTL. Within this region, we identified 23 candidate genes associated with LA biosynthesis, most notably *Glyma*.*13G112700*, which encompasses 322 significant SNPs and encodes *GmKAS2*, a key enzyme in the FA biosynthesis pathway that directly influences LA accumulation.

A genomic region significantly associated with LNA biosynthesis has been precisely mapped to a 281.17 kb interval on Chr14 (chr14:45804356–46085528) (Fig. 5e). This 281,173 bp interval contained 161 SNPs with a peak association signal of -log_10_(*P*) = 21.23 and co-localized with multiple previously reported QTLs including Seed oil 43 − 5 and several Seed LNA QTLs (3–3, 10 − 2) and Seed linoleic 3–3. The region encompasses 3,381 candidate genes, including *Glyma.14G194300*, which encodes omega-3 FA desaturase (*GmFAD3*), representing a gene-rich segment of the soybean genome with potentially significant effects on FA metabolism. This gene contained 161 significant SNPs and demonstrated remarkable stability across all five tested environments and their overall average, indicating strong and consistent genetic control of LNA content regardless of environmental conditions. The *GmFAD3* locus demonstrated significant statistical associations with multiple FA traits. Our GWAS identified one SNP associated with LA content (peak value of -log₁₀(*P)* = 6.05), one SNP associated with OA content (peak value of -log₁₀(*P*) = 6.92), and notably, 161 SNPs associated with LNA content exhibiting exceptionally strong association signals (peak value of -log₁₀(*P)* = 21.23), as illustrated in Fig. 6c.

To further characterize the genetic architecture underlying FA composition, we performed detailed association analysis of lead SNPs within the major candidate regions. The analysis revealed 64 SNPs on chromosomes 10, 14, and 20 that showed significant associations with five FA components: PA, SA, OA, LA, and LNA (Table S6). The analysis revealed 141 significant associations (*P* < 0.05), with 56 of these achieving genome-wide significance (*P* < 0.001). The most significant association was identified for SNP Gm14.45938845 located on chromosome 14 (position 45938845 bp) with LNA (*P* = 3.05e-24, *R²* = 9.84%, β = −1.1386 ± 0.1092). This locus accounted for 9.84% of the phenotypic variance in LNA content, with the T allele resulting in a decrease of 1.1386% points for each allele copy. The SNP exhibiting the most significant effect size was Gm10.50966041 (chr10:50966041) for OA (β = −4.3695, *P* = 3.96e-09), highlighting a considerable allelic influence on FA biosynthesis. The greatest proportion of variance explained was noted for Gm14.45938845 influencing LNA (*R²* = 9.84%), indicating a significant QTL for this characteristic. PA demonstrated 33 significant associations, yielding an average *R²* of 1.12% and a maximum effect size of 0.4430. Additionally, SA revealed 29 significant associations, with an average *R²* of 0.89% and a maximum effect size of 0.3138. A total of 25 notable associations were identified with OA, featuring an average *R²* of 1.89% and a maximum effect size of 4.3695. LA demonstrated 25 notable associations, featuring an average *R²* of 1.86% and a maximum effect size of 3.8094. LNA revealed 29 notable associations, with an average *R²* of 1.74% and a maximum effect size of 1.1386. Also, the top10 Associated SNPs with FA traits are presented in Table 2.

**Table 2:** Top lead SNPs and associated genetic parameters for fatty acid composition in soybean candidate regions

SNP	Chr.	POS	Alleles	Trait	MAF	Effect	SE	P_value	*R*^2^ (%)
Gm14.45938845	14	45,938,845	T/C	LNA	0.44	−1.14	0.11	3.05e^− 24^	9.84
Gm14.45938386	14	45,938,386	C/T	LNA	0.43	−1.06	0.11	6.61e^− 22^	8.87
Gm14.45939266	14	45,939,266	T/C	LNA	0.30	−0.65	0.08	1.63e^− 17^	7.02
Gm10.50971090	10	50,971,090	A/T	LA	0.41	1.73	0.23	1.09e^− 13^	5.39
Gm10.50966010	10	50,966,010	G/T	LA	0.41	1.72	0.23	1.70e^− 13^	5.31
Gm10.50971090	10	50,971,090	A/T	OA	0.41	−2.05	0.28	1.86e^− 13^	5.29
Gm10.50966010	10	50,966,010	G/T	OA	0.41	−2.05	0.28	2.44e^− 13^	5.24
Gm14.45939266	14	45,939,266	T/C	OA	0.30	1.45	0.21	2.46e^− 11^	4.37
Gm10.50965060	10	50,965,060	T/C	LA	0.40	1.44	0.22	4.64e^− 11^	4.25
Gm10.50964212	10	50,964,212	G/A	LA	0.39	1.43	0.22	6.27e^− 11^	4.20

Effect estimated additive effect size (β); SE, standard error of the effect estimate; R²(%), proportion of phenotypic variance explained by each SNP.

*SNP* Single nucleotide polymorphism, *Chr*. Chromosome, *POS* Physical position (base pairs) based on the soybean reference genome Wm82.a2.v1, Alleles, biallelic variants at each locus, Trait, fatty acid component (*PA * Palmitic acid, *SA* Stearic acid, *OA* Oleic acid, *LA * Linoleic acid, *LNA *Linolenic acid), *MAF* Minor allele frequency Effect

### Haplotype diversity and association analysis of candidate genes regulating FA biosynthesis in soybean

Haplotype analysis was conducted on three candidate genes to clarify the molecular architecture associated with variations in FA composition. The genes *Glyma.10G291700* (*GmKCS21*), *Glyma.20G111000* (*GmFAD2*), and *Glyma*.*14G194300* (*GmFAD3*) were selected due to their statistical significance, consistent detection across various environments, and recognized enzymatic roles in acyl-chain modification pathways. Sequence analysis identified unique haplotype structures within these genes, indicating varying levels of genetic diversity that may have functional consequences for FA biosynthesis.

To validate the genetic architecture that supports our haplotype definitions, we conducted LD analysis encompassing all polymorphic sites within the three candidate genes (Table S7 and Fig. S3). The LD structure demonstrated clear patterns that corroborate our haplotype block definitions. In the case of *GmKCS21* located on chromosome 10, the single SNP identified at position 50,965,060 exhibited no significant linkage disequilibrium with variants present in other genes, thereby affirming its independent segregation. For *GmFAD3* gene located on chromosome 14 exhibited significant LD with a *r²* value ranging from 0.26 to o.88 among its three SNPs at positions 45,938,386, 45,938,845, and 45,939,266, thereby confirming their classification as a singular haplotype block. The *GmFAD2* gene located on chromosome 20 displayed a complex LD pattern, characterized by weak to strong LD (*r²* = 0.22–0.9) among its seven polymorphic sites, which are situated between positions 35,312,572 and 35,323,990. This pattern aligns with historical recombination events that have resulted in the seven identified haplotypes. It is noteworthy that no substantial linkage disequilibrium was observed between genes situated on distinct chromosomes, thereby affirming their independent inheritance and validating the necessity for separate haplotype analyses for each gene.

**Fig. 7:**
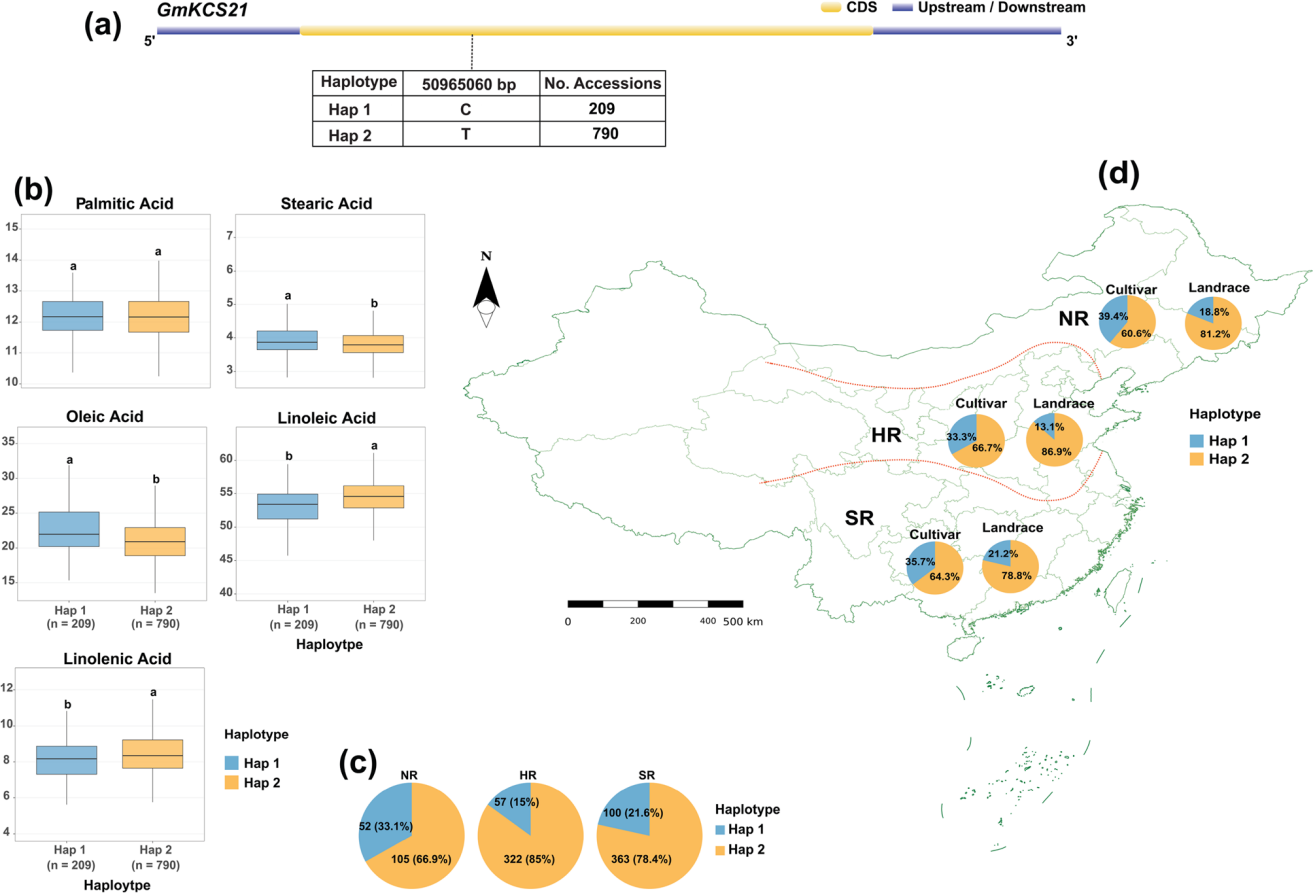
Haplotype analysis of *GmKCS21* in soybean accessions. **a** Gene structure of *GmKCS21* showing coding sequence (CDS) and upstream/downstream regions, with detailed information on two haplotypes (Hap 1: 209 accessions; Hap 2: 790 accessions) at position 50,965,060 bp. **b** Variation in FA content between the two haplotypes. Different letters above boxplots indicate statistically significant differences. **c** Distribution of haplotypes among three Chinese regions: *NR* Northern Region, *HR* Huang-Huai-Hai Region, and *SR* Southern Region. **d** Map of China showing the geographical boundaries of the three regions with pie charts indicating the distribution of haplotypes within cultivars and landraces across these regions

Allelic combinations were assessed by analyzing FA composition across soybean germplasm and their association with natural variations in the three candidate genes. In *GmKCS21*, a single synonymous variant with T/C alleles at position 50,965,060 bp defined two distinct haplotypes (Hap 1 “C” and Hap 2 “T”) (Fig. 7a). Statistical analysis revealed significant associations between *GmKCS21* haplotypes and FA composition (Fig. 7b). PA content showed no significant difference between haplotypes (*P* > 0.05), with Hap 1 and Hap 2 displaying statistically equivalent levels of 12.21% and 12.19%, respectively. However, the remaining FAs exhibited highly significant haplotype-dependent variation. Soybean accessions carrying Hap 2 demonstrated significantly lower SA content (3.83% vs. 3.92%; *P* = 5.35 × 10⁻³) and OA content (21.16% vs. 22.79%; *P* = 1.35 × 10⁻⁸) compared to Hap 1. Conversely, Hap 2 was associated with significantly elevated levels of polyunsaturated FAs, including LA (54.37% vs. 52.93%; *P* = 1.9 × 10⁻⁹) and LNA (8.46% vs. 8.15%; *P* = 5.81 × 10⁻⁴).

The analysis of the geographic distribution of *GmKCS21* haplotypes revealed distinct frequency patterns across three regions in China: NR, HR, and SR, as shown in Fig. 7c. The data demonstrates a clear predominance of Hap 2 across all surveyed regions. In NR, Hap 2 constituted 66.9% of the accessions, while Hap 1 represented 33.1%. This disparity became more pronounced in HR, where Hap 2 exhibited substantially higher prevalence at 85% compared to Hap 1 at only 15%. Similarly, SR maintained this trend with Hap 2 accounting for 78.4% of accessions versus 21.6% for Hap 1. Chi-square analysis demonstrated that haplotype frequencies differed significantly among the three geographical regions (χ² = 22.18, *P* = 1.53 × 10⁻⁵), indicating a significant association between haplotype and region.

Distinct haplotype frequency patterns emerged when comparing cultivars and landraces across three Chinese geographical regions (Fig. 7d). Landraces consistently showed higher proportions of Hap 2 than cultivars across all regions. In NR, cultivars exhibited 39.4% Hap 1 and 60.6% Hap 2, while landraces showed 18.8% Hap 1 and 81.2% Hap 2. Similarly, in HR, cultivars contained 33.3% Hap 1 and 66.7% Hap 2, whereas landraces demonstrated 13.1% Hap 1 and 86.9% Hap 2. In SR, the pattern continued with cultivars showing 35.7% Hap 1 and 64.3% Hap 2, compared to landraces with 21.2% Hap 1 and 78.8% Hap 2. Chi-square analysis indicated no significant association between haplotype distribution and accession type (χ² = 1.71, *P* = 0.1913).

Furthermore, regional variation in FA compositions was observed across Chinese geographical regions and accession types for two *GmKCS21* haplotypes (Fig. S4). For PA, region-specific patterns emerged: Hap 2 showed higher content in NR (12.40% vs. 12.14%) and HR (12.39% vs. 12.22%), but lower content in SR (11.94% vs. 12.24%). SA was consistently higher in Hap 1 across all regions, with differences most substantial in SR (3.84% vs. 3.72%). For OA, a consistently higher level was recorded by Hap 1 across all regions, with the largest difference in NR (25.16% vs. 22.88%), and significant in both cultivars and landraces. For LA, Hap 2 showed higher content in HR and SR but lower in NR, with significant effects in both germplasm types. Also, LNA displayed region-specific patterns with Hap 1 higher in NR but Hap 2 higher in HR and SR. Between accession types (Fig. S5), PA showed no significant differences between haplotypes in either cultivars or landraces, whereas SA content differed significantly only in landraces (Hap 1: 3.89%, Hap 2: 3.81%). OA was significantly higher in Hap 1 for both cultivars (24.85% vs. 23.38%) and landraces (22.50% vs. 20.93%). On contrast, LA showed the opposite pattern, with Hap 2 significantly higher in both cultivars (52.66% vs. 51.38%) and landraces (54.54% vs. 53.15%), while LNA differed significantly only in landraces, with Hap 2 higher (8.56% vs. 8.27%).

**Fig. 8:**
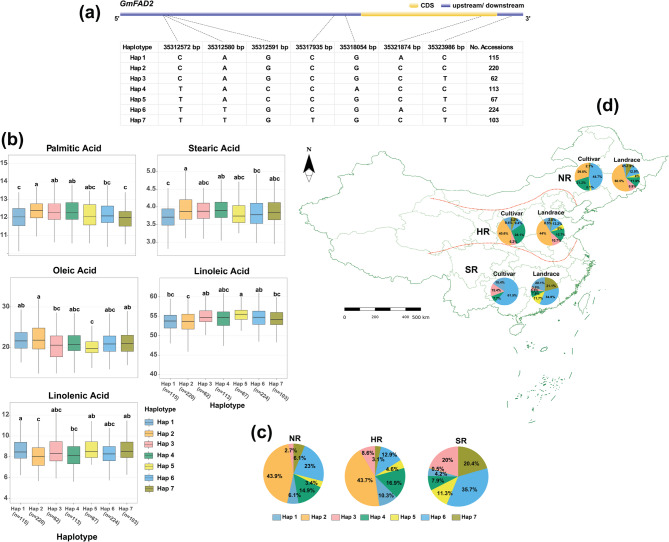
Haplotype analysis of *GmFAD2* in soybean accessions. **a** Gene structure of *GmFAD2* showing coding sequence (CDS) and upstream/downstream regions, with detailed information on seven haplotypes (Hap 1: 115 accessions, Hap 2: 220 accessions, Hap 3: 62 accessions, Hap 4: 113 accessions, Hap 5: 67 accessions, Hap 6: 224 accessions, and Hap 7:103 accessions. **b** Variation in FA content between the seven haplotypes. Different letters above boxplots indicate statistically significant differences. **c** Distribution of haplotypes among three Chinese regions: *NR* Northern Region, *HR* Huang-Huai-Hai Region, and *SR* Southern Region. **d** Map of China showing the geographical boundaries of the three regions with pie charts indicating the distribution of haplotypes within cultivars and landraces across these regions

For *GmFAD2*, sequence analysis revealed a complex polymorphic profile spanning genomic positions 35,312,572 to 35,323,986 bp, with seven non-synonymous substitutions identified. Six variants occurred in the upstream/promoter region, while one was located within the coding sequence, suggesting potential impacts on both transcriptional regulation and protein function. These polymorphisms segregated into seven distinct haplotypes: Hap 1 “CAGCGAC”, Hap 2 “CAGCGCC”, Hap 3 “CAGCGCT”, Hap 4 “TACCACC”, Hap 5 “TACCGCT”, Hap 6 “TTGCGAC”, and Hap 7 “TTGTGCT” (Fig. 8a). The results showed significant associations between those seven *GmFAD2*-variants and FA profiles in the examined soybean germplasm (Fig. 8b). Notably, Hap 2 exhibited the highest mean concentrations of PA (12.37%; *P* = 3.24 × 10⁻⁷), SA (3.95%; *P* = 1.40 × 10⁻⁵), and OA (22.11%; *P* = 1.53 × 10⁻⁵), while simultaneously displaying the lowest levels of LA (53.48%) and LNA (8.09%). Conversely, Hap 5 demonstrated an inverse pattern, with the lowest OA content (19.96%) but the highest LA concentration (55.39%; *P* = 2.36 × 10⁻⁶) and among the highest LNA levels (8.68%; *P* = 9.11 × 10⁻⁶). In contrast, Hap 1 showed the lowest PA (11.99%) and SA (3.71%) contents, while maintaining the highest LNA concentration (8.68%).

The geographic distribution analysis of *GmFAD2* haplotypes revealed striking regional variation patterns across the three Chinese regions as illustrated in Fig. 8c. A general north-to-south transition trend shows Hap 2 dominating NR populations but progressively decreasing southward, while Hap 6 and Hap 7 increase in prevalence moving south. In the NR, Hap 2 exhibited the highest frequency at 43.9%, while Hap 7 showed the lowest representation at merely 2.7%. HR maintained Hap 2 as the most prevalent haplotype at 43.7%, with Hap 1 representing the lowest frequency at 3.1%. In SR, a dramatic shift occurred with Hap 6 becoming the most abundant haplotype at 35.7%, while Hap 2 showed the lowest frequency at just 0.5%, a remarkable reversal of its dominance in NR. Chi-square analysis revealed a highly significant association between haplotype distribution and geographical region (χ² = 337.61, *P* = 5.74 × 10⁻⁶⁵), indicating strong regional structuring of *GmFAD2* haplotypes across Chinese soybean growing areas.

Also, distinct haplotype frequency patterns were observed when comparing cultivars and landraces across the three geographical regions of China (Fig. 8d). In NR, cultivars predominantly contained Hap 6 (44.7%), Hap 2 (29.8%), and Hap 4 (21.3%), while landraces showed strong preference for Hap 2 (50.5%) with lower representation of Hap 6 (12.9%). In HR, both cultivars and landraces exhibited Hap 2 as the dominant haplotype, though at different frequencies (40.6% in cultivars vs. 44% in landraces). Cultivars also showed a substantial presence of Hap 4 (28.1%), while the remaining haplotypes were more evenly distributed in both groups. In SR, Hap 6 was the predominant haplotype in both cultivars and landraces, though at markedly different frequencies (61.5% in cultivars vs. 34.9% in landraces). Additionally, the landrace population displayed greater haplotype diversity in this region compared to cultivars. Chi-square analysis indicated a significant association between haplotype distribution and accession type (χ² = 28.88, *P* = 6.4 × 10⁻⁵).

Significant regional variation in FA profiles was observed among the seven *GmFAD2* haplotypes across three growing regions (Fig. S6). In NR, Hap 5 and Hap 4 showed the highest PA contents (12.90% and 12.61%), while Hap 2 maximized SA accumulation (4.19%). OA and LA showed no significant inter-haplotype differences in NR despite numerical variations, while LNA peaked in Hap 6 (7.68%). In HR, Hap 2 exhibited maximum PA (12.52%) with Hap 7 showing minimum (11.91%). SA levels remained statistically uniform. OA content varied significantly, with the highest levels in Hap 7 (23.22%) and Hap 1 (22.42%) compared to Hap 3 (19.51%) and Hap 5 (19.45%). LA accumulation was the highest in Hap 5 (56.15%) and Hap 3 (55.39%), while LNA peaked in Hap 3 (8.89%). In SR, both PA and SA showed no significant inter-haplotype differences, while OA exhibited modest variation with Hap 5 showing the lowest content (19.75%). LA was the highest in Hap 5 (55.49%), and LNA accumulation remained consistent across all SR haplotypes.

The seven *GmFAD2* haplotypes revealed distinct FA distribution patterns between cultivars and landraces (Fig. S7). In cultivars, PA (11.90–12.54.90.54%) and SA (3.64–4.39%) remained relatively stable across haplotypes, while OA levels were significantly elevated in Hap 5 (27.48%) and Hap 7 (27.50%). These same haplotypes showed corresponding reductions in LA (48.21% and 49.62%, respectively), indicating a metabolic shift. LNA content varied from the highest in Hap 1 (8.72%) to the lowest in Hap 7 (6.97%). Landraces demonstrated different trends, with PA peaking in Hap 2 (12.38%) and Hap 3 (12.40%) while reaching minimums in Hap 1 (11.99%) and Hap 7 (12.00%). SA was the highest in Hap 2 (3.91%) and the lowest in Hap 1 (3.71%). OA reached maximum levels in Hap 1 (21.93%) and Hap 2 (21.77%), contrasting with its minimum in Hap 5 (19.85%). LA was most abundant in Hap 5 (55.49%), significantly higher than in Hap 1 and Hap 2 (both ~ 53.7%). LNA remained consistent across landrace haplotypes, with a slight elevation in Hap 5 (8.70%). Comparatively, cultivars contained higher OA levels than landraces, particularly evident in Hap 5 (27.48% vs. 19.85%) and Hap 7 (27.50% vs. 21.59%), while landraces generally exhibited higher LA and LNA concentrations.

**Fig. 9:**
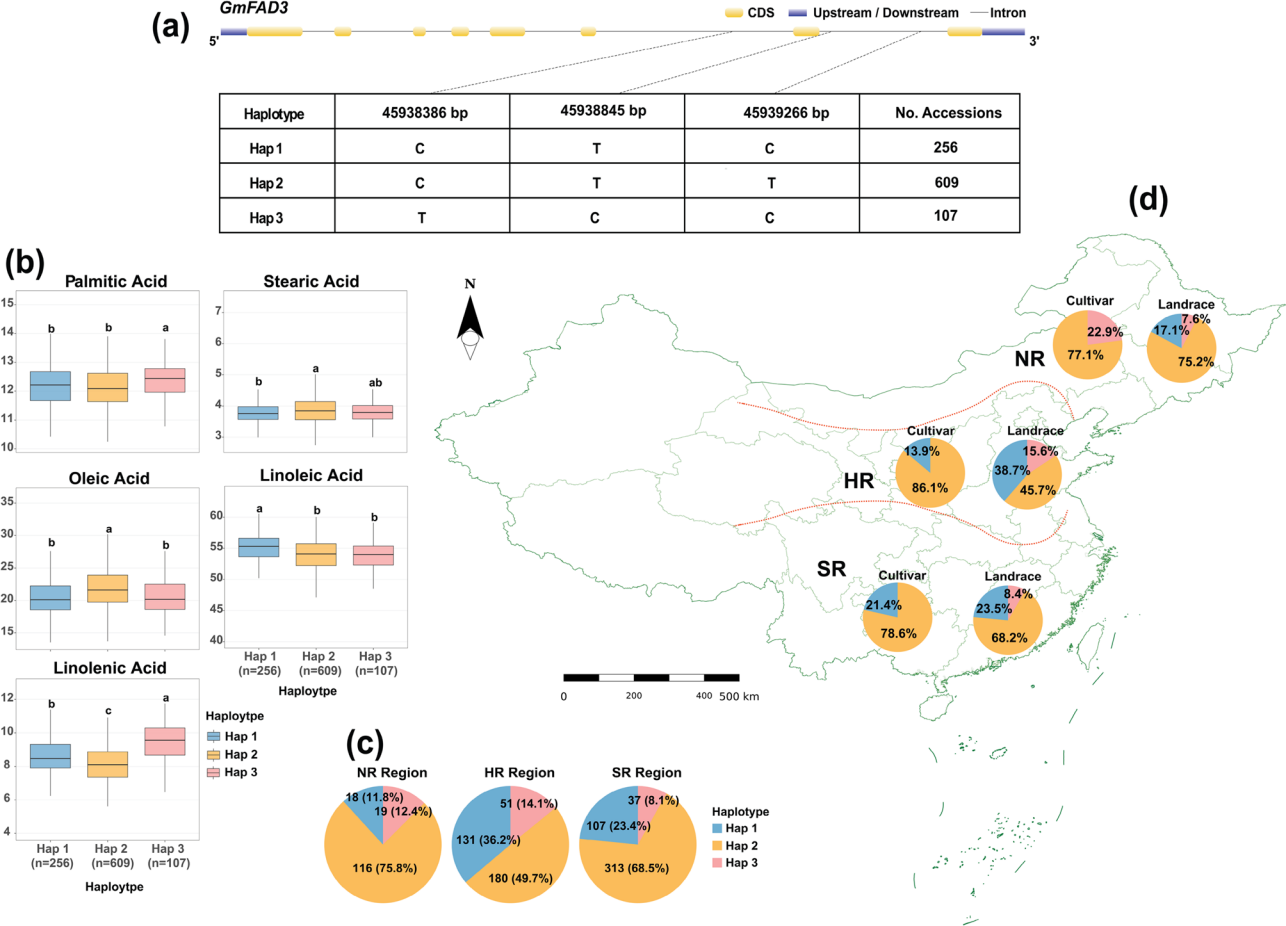
Haplotype analysis of *GmFAD3* in soybean accessions. **a** Gene structure of *GmFAD3* showing coding sequence (CDS) and upstream/downstream regions, with detailed information on seven haplotypes (Hap 1: 256 accessions, Hap 2: 609 accessions, Hap 3: 107 accessions. **b** Variation in FA content between the two haplotypes. Different letters above boxplots indicate statistically significant differences. **c** Distribution of haplotypes among three Chinese regions: *NR* Northern Region, *HR* Huang-Huai-Hai Region, and *SR* Southern Region. **d** Map of China showing the geographical boundaries of the three regions with pie charts indicating the distribution of haplotypes within cultivars and landraces across these regions

For *GmFAD3*, sequence analysis revealed three SNPs within the coding region: a synonymous variant (C/T) at position 45,938,386 bp, a missense variant (T/C) at position 45,938,845 bp, and another synonymous variant (T/C) at position 45,939,266 bp (Fig. 9a), which formed three distinct haplotypes: Hap 1 “CTC”, Hap 2 “CTT”, and Hap 3 “TCC”. The phenotypic effects of the identified haplotypes showed significant associations between *GmFAD3* haplotypes and FA compositions (Fig. 9b). PA content differed significantly among haplotypes (*P* = 7.18 × 10⁻⁴), with Hap 3 exhibiting the highest mean concentration at 12.42%, compared to Hap 1 and Hap 2, which displayed statistically equivalent levels of 12.21% and 12.14%, respectively. SA content was significantly elevated in Hap 2 (3.88%) compared to Hap 1 (3.77%; *P* = 7.15 × 10⁻⁴). OA abundance was significantly greater in Hap 2 (22.08%) than in both Hap 1 (20.58%) and Hap 3 (20.56%; *P* = 1.12 × 10⁻¹⁰). LA levels were significantly higher in Hap 1 (54.90%) compared to Hap 2 (53.77%) and Hap 3 (53.72%; *P* = 2.44 × 10⁻⁷). LNA content exhibited highly significant variation across all three haplotypes (*P* = 1.66 × 10⁻²⁸), with Hap 3 displaying the maximum concentration at 9.48%, while Hap 2 showed the minimum at 8.14%. For the geographic distribution of the frequencies of *GmFAD3* haplotypes across three Chinese regions, a noticeable regional patterns, with Hap 2 being the predominant haplotype across all regions but with varying proportions (Fig. 9c). In NR, Hap 2 accounted for 75.8% of accessions compared to 11.8% for Hap 1 and 12.4% for Hap 3. In HR, the distribution was more balanced with Hap 2 representing 49.7%, Hap 1 at 36.2%, and Hap 3 at 14.1%. The SR region showed Hap 2 dominance at 68.5%, with Hap 1 at 23.4% and Hap 3 at 8.1%, respectively. Chi-square analysis revealed a highly significant association between haplotype distribution and geographical region (χ² = 50.45, *P* = 2.91 × 10⁻¹⁰), indicating significant regional structuring of GmFAD3 haplotypes across Chinese soybean growing areas.

Overall, distinct haplotype frequency patterns were observed when comparing cultivars and landraces within the three geographical regions of China (Fig. 9d). Across all regions, landraces demonstrated different proportions of haplotypes compared to cultivars. In NR, cultivars exhibited 22.9% of Hap 3 and 77.1% of Hap 2, while landraces showed a higher proportion of Hap 1 at 17.1% and a slightly lower proportion of Hap 2 at 75.2%, with an additional 7.6% comprising other Hap 3. In HR, cultivars contained 13.9% Hap 1 and 86.1% Hap 2, whereas landraces showed a dramatically higher preference for Hap 1 at 38.7% versus 45.7% Hap 2, with 15.6% representing Hap 3. In SR, cultivars contained 21.4% Hap 1 and 78.6% Hap 2, while landraces exhibited 23.5% Hap 1, 68.2% Hap 2, and 8.4% Hap 3. Chi-square analysis indicated a significant association between haplotype distribution and accession type (χ² = 19.28, *P* = 6.50 × 10⁻⁵).

The three *GmFAD3* haplotypes affected soybean FA composition differently among three Chinese regions (Fig. S8). Hap 3 consistently had the greatest PA content across all regions (NR: 12.48%, HR: 12.59%, SR: 12.15%), with statistically significant variations in HR and SR. SA content varied: NR had Hap 3 (4.07%), while HR had Hap 2 (3.96%), greatly exceeding Hap 1 and 3. Regional variations in OA content were the greatest in Hap 2 (NR: 24.10%, HR: 22.50%, SR: 21.09%), with significant differences in NR and HR but not SR. Regional LA content patterns showed Hap 1 having the highest levels (NR: 53.46%, HR: 55.38%, SR: 54.55%). Hap 3 had the highest LNA content (NR: 8.26%, HR: 9.51%, SR: 10.06%), followed by Hap 1 and Hap 2. Hap 3 increased LNA content along the north-to-south gradient, with the greatest effect in SR.

Varying FA profiles were noticed between soybean cultivars and landraces owing to the effect of *GmFAD3*-derived haplotypes (Fig. S9). PA content remained similar across haplotypes in both groups (12.3–12.6% in cultivars; 11.7–12.5% in landraces), while SA showed minimal variation in cultivars but slight differences in landraces, with Hap 2 highest at 3.86%. Noticeably, OA was generally higher in cultivars than landraces, with Hap 2 highest in both groups (24.13% and 21.77%, respectively). LA varied significantly only in landraces, with Hap 1 highest (54.96%). LNA showed the greatest variation overall, with cultivar content highest in Hap 1 (8.49%) and landrace content highest in Hap 3 (9.64%), with all landrace haplotypes being statistically distinct.

## Discussion

Variations in FA composition in soybean seeds markedly affect the nutritional characteristics of soybean oil. The development of soybean varieties with an altered oil profile of saturated and unsaturated FA compositions is a key goal for certain soybean breeding initiatives. The current study assessed the seed FA concentrations in 1,550 soybean accessions, primarily sourced from China.

### Phenotypic variation and environmental influence on Fa composition

The analysis demonstrated a complex interaction between genetic factors and environmental influences on soybean FA profiles. For example, LA demonstrated notable stability despite high levels, suggesting strong genetic control over Δ12 desaturase activity. This contrasts with the considerable environmental variability observed in OA content, supporting the findings of Hudson & Hudson [38], and highlighting the complex regulatory processes involved in lipid biosynthesis. This can be interpreted by the negative correlation between LA and OA, which indicates a critical metabolic trade-off within the FA biosynthesis pathway, highlighting substrate competition in *FAD2*-mediated desaturation processes [39]. Also, the significant variability in LNA content across different environments underscores the sensitivity of ω−3 FA biosynthesis to environmental factors, which is consistent with the findings of Román et al. [40], which indicated that the expression of the *FAD3* gene is influenced by temperature. Such notable differential environmental responses observed between LA and LNA indicate distinct regulatory mechanisms governing Δ12 and Δ15 desaturases in soybean, documenting the findings by Zhang et al. [41]. These identified complex networks of FA metabolic interactions were also verified through our path analysis characterized by differing strengths of both positive and negative pathways. Collectively, these findings suggest that FA metabolism operates as an integrated system, rather than through isolated metabolic routes, providing important implications for breeding strategies focused on optimizing oil quality traits in various growing conditions and under changing climate scenarios.

### Candidate genes and genomic regions associated with Fa traits

Although genes like *GmFATB1A*, *GmFAD2*, and *GmFAD3* have been previously noted in soybean FA biosynthesis, this study introduces several important advancements. Our analysis of 1,550 accessions, significantly larger than previous GWAS studies (e.g., 194 accessions in Zhao et al. [26]), led to the identification of *GmKCS21* as a novel locus with pleiotropic effects on multiple FAs, thereby expanding the known regulatory network. In addition to identifying genes, we explored remarkable haplotype diversity, uncovering seven haplotypes for *GmFAD2*, three for *GmFAD3*, and two for *GmKCS21*, which highlights the specific allelic variants responsible for phenotypic variation. Our evaluation across five growing conditions and three latitudinal regions (NR, HR, SR) revealed environment-specific QTL signals and genotype-by-environment interactions that previous studies did not capture. The systematic comparison between cultivars and landraces highlighted how modern breeding has altered FA genetic architecture, while also uncovering valuable allelic diversity retained in traditional germplasm. The measurement of haplotype effects specific to regions shows that effective breeding strategies need to be customized for different geographical areas instead of being one-size-fits-all. The integrated findings provide actionable breeding recommendations featuring specific haplotype combinations for targeted oil profiles (e.g., *GmFAD2*-Hap 2 for > 70% OA; *GmFAD3*-Hap 2 with *GmFAD2*-Hap 5 for < 3% LNA), shifting our understanding from gene identification to practical, region-adapted selection strategies.

The present study identified multiple genomic regions linked to various FAs, elucidating the intricate genetic network that governs FA biosynthesis in soybean [42]. Two significant regions were identified on Chr5 and 17 linked to PA content, including *Glyma.05G012300* (*GmFATB1A*), which encodes a chloroplast-localized palmitoyl-acyl carrier protein thioesterase and exhibits a significant correlation with PA content in various environments, underscoring the essential function of palmitoyl-acyl carrier protein thioesterases in the regulation of saturated FA biosynthesis. This thioesterase specifically hydrolyzes the thioester bond of acyl-ACP in FA biosynthesis, releasing free PA and SA from the plastidial FA synthase complex. Also, comprehensive functional validation studies substantiate the critical role of *GmFATB1A* in the regulation of saturated FAs. For instance, Carrero-Colón and Hudson [43] exploited a *fatb1a* mutant identified a mutant allele characterizing *GmFATB1A* that led to early protein termination, resulting in a reduction of seed PA to 5.5%. Also, Ma et al. [44] found that single *fatb1a* and *fatb1b* mutants decreased leaf PA and SA by 11–21%, while double mutants showed more significant reductions (42% PA, 35% SA).

Noteworthy, the association of *GmKCS21* with various FAs (OA, LA, and SA) illustrates the interconnected nature of the FA metabolic network, aligning with previous findings indicating that disruptions in elongation pathways can influence multiple FA species [39]. Importantly, the identification of *GmFAD2* as significantly associated with both OA and LA content underscores its critical role in regulating the balance between monounsaturated and polyunsaturated FAs. The consistency of this association across various environments (B17, B18, and H17) corresponds with findings that *FAD2* variants significantly contribute to phenotypic variation in oil composition among different soybean germplasm [45, 46]. The elevated association signal for OA relative to LA (peak values of 8.82 versus 8.48) indicates that genetic variation in *GmFAD2* may have a more direct effect on substrate levels than on product accumulation.

The robust association signal for *GmFAD3* with LNA content (peak value of 21.23) suggests a significant locus regulating omega-3 FA accumulation, aligning with prior research [26, 38]. The consistency of this association across all five environments highlights the genetic robustness of this locus, positioning it as an optimal candidate for marker-assisted selection. Additionally, the pleiotropic effects of *GmFAD3* on LA and OA content exemplify the interconnectedness of the FA biosynthetic network. This enzyme can desaturase LA (18:2, Omega6) to LNA (18:3, Omega3), as overexpression of *FAD3* can increase the proportion of LNA [47, 48]. Our results have been supported by a prior GWAS study of a natural population consisting of 194 distinct soybean accessions, which identified *Glyma.14G194300* (*GmFAD3*) as a regulator of LNA accumulation [26]. Consequently, our research substantiates the significance of *GmFAD3* in FA metabolism. Moreover, another prior investigation has also confirmed and mapped the *Glyma.14G194300*, acting as *GmFAD3A* gene, and its linked QTLs, encompassing seed linoleic 3–3 from the RG10 × OX948 population and Linolen-PO (*FAD3A*) validated in the PI 361088B × OX948 population, to this gene [49]. The confirmed association between linolenic acid QTL and *GmFAD3A*, substantiated across distinct populations, coupled with the creation of *GmFAD3* gene-specific markers, provides essential resources to enhance and accelerate breeding initiatives aimed at low linolenic acid soybean varieties.

### Linkage disequilibrium patterns reflect selection history and genetic diversity

Our LD analysis indicated a genome-wide decay distance of around 97 kb, aligning with earlier findings in cultivated soybean [34, 35]. Using this decay pattern, we implemented a ± 100 kb window around lead SNPs to pinpoint candidate genes within high-LD blocks (*r²* ≥ 0.8), offering strong evidence for gene assignments associated with FA biosynthesis.

Importantly, LD patterns varied significantly among germplasm types, providing valuable insights into genetic diversity and selection intensity. Enhanced cultivars showed a slower rate of LD decay and longer haplotype blocks in comparison to landraces, indicating a decrease in genetic diversity as a result of rigorous artificial selection in contemporary breeding practices [35]. This pattern aligns with selective sweeps and genetic bottlenecks linked to domestication and cultivar development. In contrast, landraces, especially those from the SR of China, exhibited rapid LD decay, suggesting significantly greater genetic diversity and reduced historical selection pressure [34]. Among geographic groups, USA accessions exhibited the most extensive LD, indicating the narrow genetic base of North American soybean resulting from limited ancestral introductions [50].

The differential LD patterns carry significant implications for breeding and gene discovery. The extended linkage disequilibrium in enhanced cultivars aids in association mapping, yet it diminishes the resolution necessary for fine-mapping causal variants. On the other hand, the swift LD decay observed in landraces provides enhanced mapping resolution and underscores their significance as reservoirs of genetic diversity for expanding the genetic foundation of contemporary breeding programs. By integrating these population-specific LD patterns into our candidate gene identification approach, we have bolstered the reliability of gene assignments while considering the diverse genetic architecture present in subpopulations.

### Haplotype diversity and geographic distribution patterns

The haplotype diversity of the three significant candidate genes: *GmKCS21*, *GmFAD2*, and *GmFAD3* was further examined. Although variation in *GmKCS21* is synonymous, two distinct haplotypes exhibited significant associations with FA profiles, indicating possible effects on mRNA stability, translation efficiency, or splicing regulation [51]. Synonymous variants may influence enzyme kinetics by altering co-translational folding, which could elucidate their impact on metabolic flux [52, 53]. Furthermore, the analysis of geographic distribution indicated a predominance of *GmKCS21*-Hap 2 across all surveyed regions in China, exhibiting a notably higher frequency in HR compared to NR and SR. This observation suggests a potential selection pressure associated with environmental adaptation [54]. Also, the elevated prevalence of *GmKCS21-*Hap 2 in landraces relative to cultivars indicates that traditional agricultural practices may have preferentially selected this haplotype owing to its adaptive benefits. Ultimately, the region-specific impacts of *GmKCS21* haplotypes on FA composition provide additional evidence for mechanisms of environmental adaptation. The observed variation in PA content among regions, where *GmKCS21-*Hap 2 exhibits elevated levels in NR and HR but diminished levels in SR, illustrates intricate genotype-by-environment interactions that influence FA metabolism. This phenomenon aligns with the findings of Le Luyer et al. [55], which documented differential expression of FA biosynthesis genes along latitudinal gradients. The pronounced influence of haplotypes on SA content in landraces, compared to cultivars, indicates that contemporary breeding practices may have introduced elements that obscure the effects of the *GmKCS21* variant [56].

The polymorphic profile of *GmFAD2*, characterized by seven non-synonymous substitutions that delineate seven distinct haplotypes, exemplifies the complex genetic regulation of FA desaturation. The identification of six variants within the upstream/promoter region indicates possible mechanisms of transcriptional regulation, whereas the coding variant may have a direct effect on enzyme functionality. This combination elucidates the significant phenotypic effects observed, with *GmFAD2*-Hap 2 favoring the accumulation of saturated and monounsaturated FA, whereas *GmFAD2-*Hap 5 promotes the accumulation of polyunsaturated FAs. The distinct regional distribution patterns of *GmFAD2* haplotypes, characterized by the predominance of *GmFAD2-*Hap 2 in NR and its near absence in SR, indicate adaptive evolution in response to environmental gradients. Noteworthy, the north-to-south transition corresponds with the findings of Wu et al. [57], indicating that lipid desaturation requirements differ according to temperature regimes. The differential distribution in *GmFAD2* haplotypes between cultivars and landraces illustrates the influence of selection pressures, with cultivars in NR predominantly exhibiting *GmFAD2-*Hap 6, whereas landraces preferentially display *GmFAD2-*Hap 2. The significantly elevated OA content in cultivars compared to landraces, especially in *GmFAD2-*Hap 5 and *GmFAD2-*Hap 7, aligns with contemporary breeding initiatives aimed at producing high-oleic varieties [58].

The notable association between *GmFAD3*-derived haplotypes and LNA accumulation, particularly with *GmFAD3*-Hap 3 demonstrating the highest levels, reinforces the gene’s primary regulatory role in omega-3 FA biosynthesis [38]. Analysis of geographic distribution indicated that *GmFAD3*-Hap 2 is the predominant variant across all regions. The observed balanced distribution in HR relative to NR indicates that mid-latitude environments may preserve a higher genetic diversity concerning omega-3 desaturation capacity. The significant variation in haplotype distribution observed between cultivars and landraces in HR indicates substantial artificial selection throughout contemporary breeding practices. The consistent pattern of *GmFAD3-*Hap 3 maximizing LNA content across all regions, with effects intensifying along the north-to-south gradient, indicates that elevated temperatures may enhance the phenotypic expression of these genetic variants. This observation is consistent with the findings of Menard et al. [59], which demonstrate that temperature influences *FAD3* expression and activity.

### Implications for soybean breeding and genetic improvement

The identifications of these haplotypes offer valuable targets for implementing marker-assisted selection in soybean breeding initiatives. To develop high-oleic cultivars with improved oxidative stability for food industry applications, breeders should focus on selecting *GmFAD2*-Hap 2, which has shown a notable increase in OA content (> 25%) while decreasing polyunsaturated FA levels. This combination of haplotypes would be especially beneficial for creating cooking oils that offer a longer shelf life and enhanced frying capabilities. On the other hand, for low-linolenic varieties aimed at industrial applications that demand oxidative stability, the combination of *GmFAD3*-Hap 2 with *GmFAD2*-Hap 5 could result in LNA levels under 3%, while still preserving sufficient LA content to meet nutritional needs. The observed distribution patterns specific to the region indicate that *GmFAD2*-Hap 2 would be most advantageous in NR breeding programs, whereas SR could gain from incorporating this allele from SR germplasm.

The combination of GWAS results with thorough haplotype analyses in this study provides a comprehensive understanding of the genetic architecture that influences FA composition in soybean. The ongoing identification of *GmKCS21*, *GmFAD2*, and *GmFAD3* highlights their significance as crucial factors in FA profiles, while haplotype analysis elucidates the particular allelic variants that influence phenotypic variation. In light of these findings, we suggest a structured breeding strategy aimed at enhancing particular oil quality traits: (1) To achieve high-oleic varieties (> 70% OA) with improved oxidative stability for food applications, it is beneficial to combine *GmFAD2*-Hap 2 with specific GmKCS21 haplotypes while avoiding active *GmFAD3*-Hap 3 alleles to enhance monounsaturated FA accumulation; (2) For ultra-low linolenic varieties (< 1% LNA) needed for industrial applications, pairing null or low-activity *GmFAD3* alleles with *GmFAD2* variants that preserve adequate LA levels will provide the necessary stability without compromising nutrition; (3) To ensure balanced nutritional profiles, maintaining *GmFAD3*-Hap 3 while adjusting *GmFAD2* expression could optimize omega-6/omega-3 ratios for human health applications [39].

Ultimately, the region-specific effects of haplotypes on FA compositions emphasize the importance of considering the environmental adaptation in soybean breeding and pinpoint that optimal haplotype selection should be tailored to specific regions rather than a universal strategy for different growing environments. The differential distribution of haplotypes between cultivars and landraces elucidates the genetic implications of contemporary breeding practices, as landraces retain significant genetic diversity, which may have been reduced through modern selection processes, which is consistent with the findings of Hyten et al. [60], who identified genetic bottlenecks in oil quality traits throughout soybean domestication.

## Conclusion

This comprehensive study of seed FA composition in 1,550 soybean accessions in five environments has substantially enhanced our understanding of the molecular basis of soybean seed FA traits. By integrating phenotypic characterization with GWAS and haplotype analysis, we identified the genetic determinants and regulatory networks that regulate FA biosynthesis and accumulation. Functional enrichment analysis has also supported their involvement in lipid synthesis pathways, while GWAS showed 110,964 significant SNP-trait associations spanning 18,841 genes on all 20 chromosomes. Our haplotype study of *GmKCS21*, *GmFAD2*, and *GmFAD3* revealed FA profile-related allelic variations and geographic distribution patterns in NR, HR, and SR in China. FA composition adaptability and selection pressures are suggested by north-to-south haplotype frequencies. We also discovered different haplotype distribution patterns in cultivars and landraces, suggesting genetic architecture changed throughout domestication and breeding. The candidate genes and haplotypes identified in this study offer promising targets for marker-assisted selection and genetic engineering, with the goal of developing soybean varieties with customized FA profiles for nutritional, industrial, and environmental applications. In particular, the identified SNP markers can be integrated into genomic selection models to increase breeding efficiency, while favorable haplotypes of *GmFAD2* and *GmFAD3* can be used to create high-oleic varieties with improved oxidative stability. Furthermore, the precise optimization of the FA profile is facilitated by the CRISPR/Cas9-mediated editing of critical regulatory regions in these candidate genes. Future functional investigations should verify these candidate genes and investigate their potential use in region-specific breeding strategies that focus on local haplotype advantages to optimize FA composition for specific end-use applications and diverse growing conditions.

## Supplementary Information

Supplementary Material 1.

## Data Availability

No datasets were generated or analysed during the current study.
